# Country progress towards the Millennium Development Goals: adjusting for socioeconomic factors reveals greater progress and new challenges

**DOI:** 10.1186/s12992-014-0067-7

**Published:** 2014-10-01

**Authors:** Robert L Cohen, Yira Natalia Alfonso, Taghreed Adam, Shyama Kuruvilla, Julian Schweitzer, David Bishai

**Affiliations:** Johns Hopkins Bloomberg School of Public Health, 615 N. Wolfe Street Suite E4622, Baltimore, MD 21205 USA; Alliance for Health Policy and Systems Research, World Health Organization, 20 Avenue Appia, 1211 Geneva, Switzerland; Partnership for Maternal, Newborn & Child Health, World Health Organization, 20 Avenue Appia, 1211 Geneva, Switzerland; Results for Development, Washington, DC USA

## Abstract

**Background:**

The health Millennium Development Goals (4, 5, 6) impose the same ambitious 2015 targets on every country. Few low-income countries are on track to reach them. Some authors have proposed country-specific targets as a more informative method by which countries can measure their progress against their potential.

**Methods:**

This paper demonstrates a supplementary approach to assess individual country progress that complements the global goals by adjusting for socioeconomic resources and prior time trends. A *minimum performance target* adjusts for time and national GDP. *Fast-track targets*, based on best-performing countries’ progress within regional and income groups, adjust for health and non-health sector factors known to affect maternal and child health.

**Results:**

Measuring by the minimum performance target, 74% and 59% of low- and middle-income countries are on track for reducing child mortality and maternal mortality, respectively, compared with 69% and 22% using global MDGs. Only 20% and 7% of low- and middle-income countries are on track for the child and maternal mortality fast-track targets.

**Conclusions:**

Supplementary targets in maternal and child health, adjusted for each country's resources and policy performance can help countries know if they are truly underperforming relative to their potential. Adjusted targets can also flag countries that have surpassed their potential, and open opportunities for learning from success.

**Funding:**

Partnership for Maternal, Newborn & Child Health and the Alliance for Health Policy and Systems Research, as part of the Success Factors Study on reducing maternal and child mortality.

**Electronic supplementary material:**

The online version of this article (doi:10.1186/s12992-014-0067-7) contains supplementary material, which is available to authorized users.

## Introduction

The Millennium Development Goals (MDGs) originate from the 2000 United Nation (UN) Millennium Declaration adopted by 189 UN member states during the Millennium Summit [1]. Health MDGs 4 and 5a had respective targets to reduce the under-5 mortality rate (U5MR, the number of deaths of children under 5 per 1,000 live births) by two thirds and the maternal mortality ratio (MMR, the number of maternal deaths per 100,000 live births) by three quarters by 2015 and to achieve universal access to reproductive health [1]. MDG 4 was partially based on observations of reductions in child mortality in three countries that achieved high rates of progress between 1960 and 1990 (China, Sri Lanka and Vietnam) [2]. This targeted improvement was then applied as a global goal to achieve similar progress worldwide from 1990–2015. The Millennium Declaration uses the collective pronoun, “We” and refers to duties to the “world’s people”, leading many to conclude that the goals and indicators were intended as motivational aspirations for the aggregate world population. Whatever the General Assembly’s original intentions, numerous reports and studies have reinterpreted the MDGs as goals applying to each individual country [3-5]. Many of these reports have noted that the vast majority of low- and middle-income countries, especially in Africa, are “off track” for meeting the MDGs despite rapid progress in some places against considerable concurrent challenges. This has led some to suggest that applying a global standard to every country may be inappropriate [6]. Country-specific targets could allow people of a given country to better assess whether they are truly underperforming given their resources and potential.

The High Level Panel on the Post-2015 development agenda and the Open Working Group on Sustainable Development reports indicate that assessment of country progress towards maternal and child health goals will continue to be part of the post-2015 landscape [7,8]. Because assessment of country progress will continue to be important after 2015, assessment tools designed specifically to compare a country’s progress to its own specific potential should be considered. Despite the great merit of the MDGs as global aspirations, global targets do not adjust for local factors that might inhibit or enhance progress. Very few sub-Saharan countries have a realistic chance to meet proposed 2030 targets for ending preventable newborn and child mortality or achieving a U5MR of 25 and a MMR of less than 70 [9,10]. This paper explores methods to supplement a single global target to demonstrate what can be learned when accounting for country factors.

### What global and country-specific targets can and can’t do

When the health MDGs are used as country-specific performance targets, the latest reports show that very few countries are on track to meet MDGs 4 and 5a, as measured by average annual percent reduction in mortality. Out of the 75 Countdown to 2015 countries (an initiative that monitors progress in the 75 countries that account for more than 95% of preventable maternal and child mortality worldwide), only 28 are on track for MDG 4 [5]. Of these, only nine are from Sub-Saharan Africa (SSA) [11]. For MDG 5a, only 20 out of the same 75 are on track, and only 6 in SSA [11]. Only 13 are on track for both goals, 3 from SSA. (Kuruvilla S, Franz-Vasdeki J, Chowdhury S et al.: “Strategies that 'High-Performing' countries used in their efforts to reduce maternal and child mortality: a multi-country, multi-method study of 'Success' factors for MDGs 4 and 5a”, forthcoming)

Using the MDGs to monitor progress and classify countries as on or off track, while understandable, can be misleading because the numerical targets were based on time trends of a few countries without attention to what could be reasonably achieved by each particular country given their available resources [12]. Off track countries can realistically be excused from attaining targets that were unachievable in the first place and LMIC policy makers can question the relevance of targets that were set without regard to local resources and challenges. The best proof of unattainability is that over 80% of low-income African countries currently appear as “off track” for both MDG 4 and 5, despite having made substantial progress. Additionally, no Latin American countries are on track for MDG5a. In settings such as these, while acknowledging the clear value of global goals, it will also be instructive to assess country performance using country-specific indicators that adjust for a country’s socioeconomic and epidemiological potential. Commentators note a harmful sense of futility when a country that is actually making acceptable progress in improving health is misclassified as off track [12,13].

Focusing on percentage reductions requires an unwarranted assumption that it is just as easy for a country with under-five mortality rate (U5MR) of 240 to bring their rate down by 160 points as it is for a country with U5MR of 120 to bring the rate down by 80 points. Easterly examined the historical record of cases where a country brought U5MR down by 66% in 25 years and found that only 11% of cases could achieve this degree of health improvement starting from a U5MR that was higher than the average for all countries in Africa in 1990 [13]. More feasible and context-specific targets are needed going forward.

### Framework

This study adjusts targets for maternal and child health for socioeconomic and policy factors that can improve and impair progress. This is an analogous approach overall to a separate analysis by Walker et al. that adjusted expectations of progress for the year 2035 based on each country’s trajectory in implementing different public practices between 1990 and 2011. Based on a model of how many lives can be saved through interventions like vaccinations, oral rehydration, and attended delivery, this approach forecasted future lives saved based on expectations of better coverage with interventions. It found that even in a best-case scenario of increased coverage of key interventions, there is only a marginal increase in the percent of countries on track for proposed 2035 targets. Our study proposes a supplemental approach that relies on the correlation between mortality and socioeconomic factors, rather than models of improvements in the coverage of specific interventions. The impacts of health intervention programs are determined by enabling factors such as a country’s economic resources and factors such as governance and accountability [14,15].

In order to explore the implications of supplementing a single global target with country-specific targets, this study develops two versions for country-specific performance targets. Countries are rated on what their performance might have looked like if work towards implementing the targets started in 2000 and was assessed in 2010. First, a *minimum performance target* adjusts expected performance based on each country’s prior health trajectory and each country’s projected economic growth. Per capita GDP growth yields resources that can be used to improve health. The relationship is imperfect—economic growth has been known to occur without the improvement of health and vice versa. It always depends on how GDP resources are deployed [16]. However, for the majority of low income countries, there is an empirical correlation between GDP growth and health improvements. GDP growth is a surrogate marker for a wide array of physical, social and institutional improvements that can support better health, so we use it along with time trends as a summary statistic to produce the minimum performance target. Meeting the minimum performance target means a country is maintaining past momentum and taking advantage of new economic resources as they become available for use in improving women’s and child’s health. Failing to meet this minimum performance target would indicate a deceleration of progress. Second, a *fast-track target* adjusts for a complete set of best policy practices that their best-performing regional neighbors were able to implement between 2000 and 2010. This *fast-track* adjusted target will be difficult to attain and countries that have achieved it should be closely examined to identify success factors.

## Methods

Target 1—the *minimum performance target*—was based on a model of how each country’s time trend and GDP growth affected mortality from 1990 to 2000. The model was used to project the respective mortality rates for each country to 2010, based on their expected rates of economic growth and yearly improvements. The statistical method fit a log-linear regression of the health indicator against a time trend and GDP per capita then applied the estimated coefficients to each country’s GDP growth to adjust each country’s expected degree of health improvement. The statistical method also adjusted for country specific fixed effects. To meet the target, a country had to simply achieve mortality improvements per dollar of their own GDP growth at the average rate of mortality improvement per dollar of growth observed in the sample of countries. Countries that did not reach the minimum performance target for U5MR and MMR by 2010 must have been achieving less health improvement per dollar of their GDP growth than the average performance for countries at similar GDP levels. In theory, if the model fit the data perfectly, half of all countries should be found to be below the average of all the countries in the sample and half would be above this average. In practice, country fixed effects and omitted factors could cause more countries to perform better than average. Countries were considered on track in 2010 for each target if their reported U5MR and MMR were lower than the respective targets, or below 40/1,000 live births for U5MR. Tests of robustness and sensitivity and other technical details are presented in the Additional file 1: Appendix.

Target 2—the *fast-track target*—was based on a model of how a set of key socioeconomic and policy factors that have been established in the scientific literature as contributing to improvements in maternal and child health, as well as how past time trends and GDP affected mortality rates. The factors used to adjust the model are shown in Table 1 and their derivation is discussed in the Additional file 1: Appendix and in a prior paper [8]. The list of independent variables is highly correlated because most are correlated with overall development. Hence an analysis of robustness found that shifting this list of variables by excluding one or adding another had a negligible impact on the country specific target and the determination of which countries were meeting their fast-track targets (Additional file 1: Appendix). The model projected mortality to 2010, but assumed that each country had maximal rates of improvement on the key socioeconomic determinants of mortality from 2000 to 2010. Maximal rates were established by the best rates of improvement in socioeconomic factors observed anywhere in the country’s UN sub-region from 2000 to 2010. To meet the fast-track target, a country’s 2000–2010 actual health improvement had to achieve the health improvement that would have occurred had it accomplished the maximum improvement rate in each of the policy variables that was observed by high-performing countries in its sub-region between 2000 and 2010.

**Table 1:** **Summary statistics for U5MR and MMR models**

**Under 5 Mortality rate model**	**Original data from 144 countries**			**Imputed data from 144 countries**			**Year**
	**Obs.**	**Mean**	**Std. Dev**	**Obs.**	**Mean**	**Std. Dev**	
Under 5 mortality rate (U5MR)	3001	68.6	58.8	3001	68.6	58.8	1990-2011
GDP per capita	3001	1955.5	2046.6	3001	1955-5	2046-6	1990-2011
Log kilowatt hours per capita	1878	6.6	1.4	3001	6.3	1.4	1990-2011
Percent urban	3001	46.0	20.4	3001	46.0	20.4	1990-2011
Log 5 year lag gov. health expenditure							1990-2011
Per capita	1678	4.2	1.2	3001	3.9	1.2	
Log odds girls primary school enrollment	1266	1.9	1.4	3001	1.8	1.3	1990-2011
Log odds of having clean water	2701	1.8	1.5	3001	1.8	1.5	1990-2011
Log odds of measles vaccine	2946	1.8	1.4	3001	1.8	1.4	1990-2011
Doctors per 100,000 sq. root	1146	10.2	6.2	3001	8.6	5.5	1990-2011
Control of corruption score	1812	−0.4	0.6	3001	−0.4	0.5	1996-2011*
**Maternal mortality rate model**	**Original data from 116 countries**			**Imputed data from 116 countries**			**Years**
	**Obs.**	**Mean**	**Std. Dev**	**Obs.**	**Mean**	**Std. Dev**	
Maternal mortality ratio (MMR)	339	235.6	287.0	339	235.6	287.0	1990-2011
GDP per capita	339	2067.1	2135.6	339	2067.1	2135.6	1990-2011
Control of corruption score	289	−0.4	0.6	339	−0.4	0.6	1996-2011*
Log odd skill birth attendant (SBA)	200	1.9	2.1	339	2.0	1.9	1990-2011
Total fertility rate (TFR) sq. root	339	1.8	0.4	339	1.8	0.4	1990-2011
Gini coefficient	114	45.8	9.0	339	44.7	7.9	1990-2011

*Data available every 2 years between ’96-’02 and every year between ’03-’11.

Multivariate regression adjusting for country-specific trends and fixed effects was used to make country-specific projections of the minimum and fast-track performance targets. The dependent variables were log transformations of U5MR and MMR corresponding to MDGs 4 and 5. For the minimum target the independent variables were GDP per capita and a time trend. For the fast-track target, independent variables were based on the United Nations Development Programme proposal that plans designed to meet the MDGs should include investments in seven “clusters” of public policy [17]. We use these clusters as a guide to group variables into priority areas that could represent a complete set of resources, together with health and socioeconomic public practices that impact child and maternal health.

Our final model included the following variables, all of which have statistical as well as theoretical and empirical support: percent of the population with access to clean water, [11,18-20] percent of children under 2 who received the measles vaccine, [11,21-23] a control of corruption index reported by the World Bank, [11,24] power consumption per capita, [20] urbanization, [18,23-26] percent of girls enrolled in primary school, [26-28] total fertility rate, [11,18,29,30] physicians per capita, [11,20,26,27,31-33] percent of births attended by a skilled birth attendant, [11,21,34] health spending per capita lagged by five years (since health spending in a given year may be endogenous to our model) [15,26,32,35,36], and Gini coefficient [21,34,37].

We excluded all countries classified as high-income by their 2000 GDP per capita (defined as $9,266 [38] in 2000 US dollars) from both analyses. We additionally excluded all European countries from the MMR analysis, since MMR in those countries is comparable to high-income countries. These criteria left 144 countries eligible for the U5MR analysis and 116 countries in the MMR analysis.

Data for outcome and independent variables of interest was extracted and compared between the following sources: WHO Global Health Observatory, [39] WHO National Health Accounts (NHA), [40] UNdata, [41] UNDP, [42] UNICEF’s Childinfo, [43] the World Bank DataBank, [44] and Demographic and Health Surveys (DHS). [45] Completeness of the dataset varied by indicator, see Table 1. For MMR data, our analysis uses the national estimates from UNICEF’s Child Info [43]. For U5MR, estimates are developed by the UN Inter-agency Group for Child Mortality Estimation (UNICEF, WHO, World Bank, UN DESA, UNDP). For those independent variables lacking complete data, simple regression imputation was used. The inclusion of imputed data had little effect on the targets for countries that had complete data. Since complete vital registration is uncommon in low income countries, the official measures of mortality are derived by supplementing household survey data about deaths in a household with demographic models. As noted by Deaton, the health experiences of low income countries are so different in composition and in the way mortality data are acquired that the analysis should not combine low and high income countries [46]. The data on mortality that we used are the exact same official measures that are used to track MDG progress by the UN. We replicated our results with Institute of Health Metrics measures and found results that were very similar. Until vital registration systems improve, there will be no other recourse but to use the mortality indicators that are on hand.

## Results

Table 2 shows that poverty and a higher starting initial mortality make it harder to reach the 66% mortality reduction target. Absolute point reductions in U5MR averaged 74 points in the lowest income countries and 16 points in the highest income countries across four GDP per capita quartiles (Table 2). However, expressed as percent reductions, the countries with the lowest income reduced U5MR by 42%, while LMIC counties with the highest incomes lowered U5MR by 51%. The population weighted percent reduction in U5MR for these 144 LMIC countries was 55% between 1990 and 2010.

**Table 2:** **MDG 4 & 5 progress observed from 1990 to 2010 by income quartile**

	**Lowest GDPpc quartile**	**Second GDPpc quartile**	**Third GDPpc quartile**	**Highest GDPpc quartile**	**LMICs**
**Under-5 mortality rate (U5MR)**					
144 countries	(2000 GDPpc range: $87 to $393)	(2000 GDPpc range: $402 to $1200)	(2000 GDPpc range: $1208 to $2512)	(2000 GDPpc range: $2753 to $8775)	(2000 GDPpc range: $87-$8775)
Mean absolute reduction in U5MR (per 1,000)	74 (−6 to 183)	33 (−18 to 82)	27 (−26 to 93)	16 (3 to 56)	38 (−26 to 183)
Mean percent reduction in U5MR (non-weighted)	42% (−6 to 70)	42% (−12 to 74)	52% (−31 to 88)	51% (16 to 80)	46.8% (−31 to 88)
Population weighted mean percent reduction in U5MR	46%	54%	59%	61%	55%
Birth cohort weighted mean percent reduction in U5MR*	41%	42%	60%	61%	40%
**Maternal mortality rate (MMR)**					
116 countries (wealth quartiles redrawn to exclude Europe)	(2000 GDPpc range: $87 to $359)	(2000 GDPpc range: $362 to $949)	(2000 GDPpc range: $972 to $2178)	(2000 GDPpc range: $2211 to $8775)	(2000 GDPpc range: $87-8775)
Mean absolute reduction in MMR (per 100,000)	386 (−180 to 1130)	219 (−120 to 820)	62 (−140 to 260)	50 (−51 to 770)	179 (−180 to 1130)
Mean non-weighted percent reduction in MMR	43% (−20 to 78)	41% (−27 to 82)	30% (−64 to 83)	27% (−86 to 93)	35% (−86 to 93)
Population weighted percent reduction in MMR	48%	64%	50%	38%	51%
Birth cohort weighted percent reduction in MMR*	48%	53%	49%	31%	48%

*Excludes Kiribati, Marshall Islands, Palua, Serbia, and Tuvalu for lack of birth cohort information.

The population weighted percent reduction in MMR for the world is 51%, during the same period. Likewise, MMR progress across the four GDP per capita quartiles shows that absolute point reductions averaged 386 points in the lowest income countries and 50 points in the highest income countries. Expressed as percent reductions, the lowest income countries lowered MMR by 43%, while the highest income countries lowered MMR by 27%.

**Figure 1:**
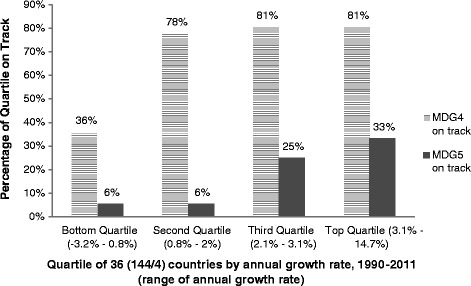
**Progress toward current MDGs 4 & 5 by annual GDP growth rate.**

Figure 1 shows that the number of countries on track to achieve MDGs 4 and 5 is much lower in the lowest-growth LMICs compared to the highest-growth group.

**Figure 2:**
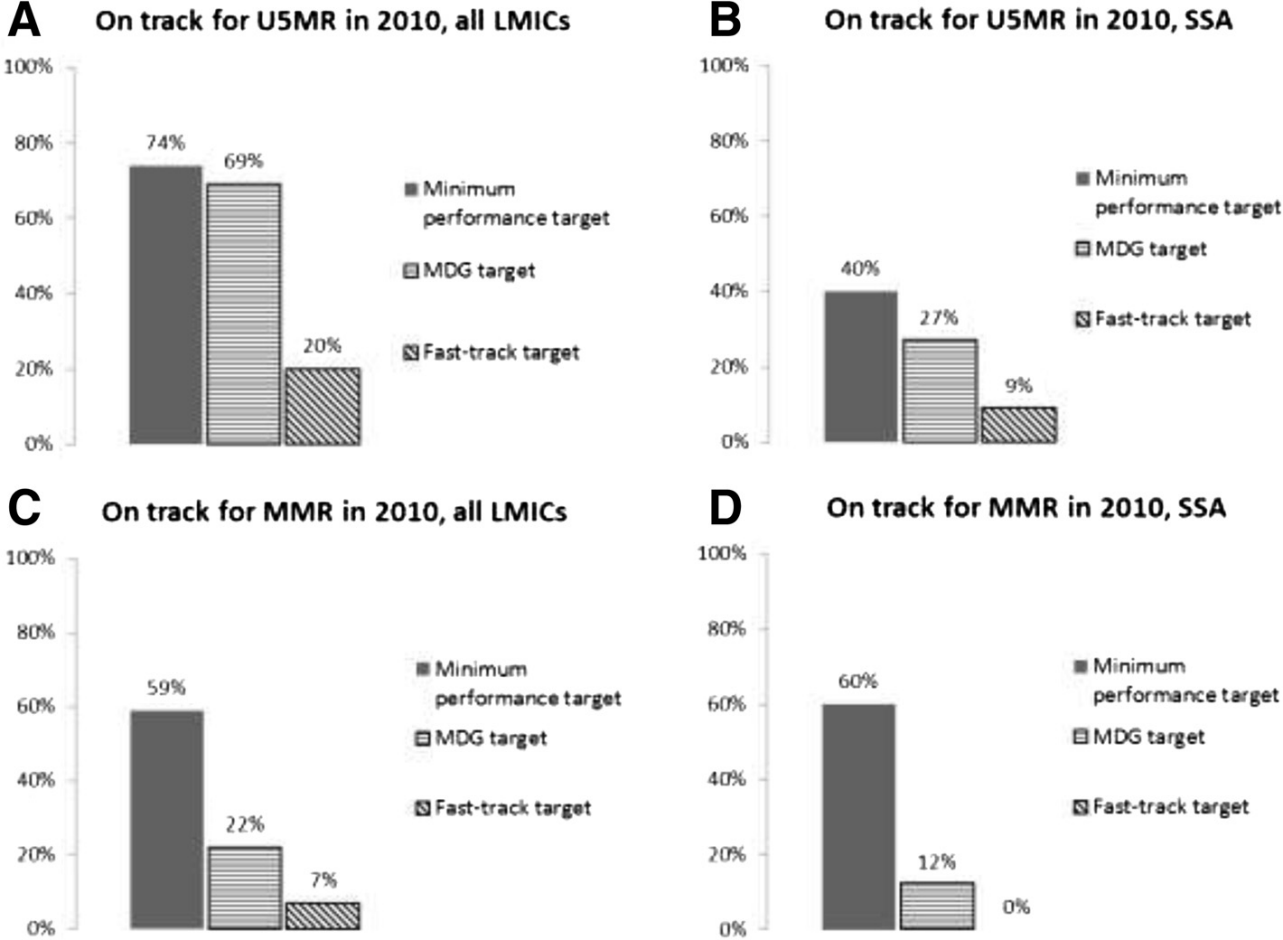
**Percent of countries on track for MDGs 4 & 5, minimum performance target, and fast-track target.** The percent of countries in each group on track by each target. **A)** U5MR, 144 LMICs. **B)** U5MR, 45 SSA countries. **C)** MMR, 116 LMICs. **D)** MMR, 42 SSA countries. MDG target is 66% reduction in U5MR or U5MR less than 40, and 75% reduction in MMR.

Table 3 shows the reported U5MR of each country in our dataset in 1990 and 2010. These can be compared to an MDG target showing what a country’s U5MR would be in 2010 if it were on a linear path to achieve a 67% reduction in the 1990 U5MR by 2015. Of 144 countries available for analysis, we find that 99 (69%) are on track by the MDG 4 target including 12 (27%) in sub-Saharan Africa. In contrast, under our U5MR *minimum performance target* 107 countries of 144 (74%) would be considered on track and 18 of 45 (40%) of sub-Saharan African countries would be considered on track (Figure 2A-B).

**Table 3:** **MDG 4: Countries’ performance based on global, minimum performance and fast-track targets listed by income group**

			**U5MR MDG target**			**U5MR aalternative 1 minimum performance target**	**U5MR alternative 2 fast-track target**	**SUMMARY OF COUNTRIES ON TRACK†**		
			**(Time projections only)**			**(Time & GDP adjusted)**	**(Time, GDP, & policy factor adjusted)**			
Income group		Country	U5MR Actual 1990	U5MR Actual 2010	*MDG 4 Target for 2010*	*2010 Target adjusted for GDP and country trajectory*	*2010 Target based on adoption of best attainable policies in the region*	On track by global MDG 4 target	On track by minimum performance target	On track by fast-track target
**LOW INCOME GROUP**										
	*	Bangladesh	138.8	48.7	64.8	57.8	47.9	✓	✓	
	*	Benin	177.3	108.8	82.7	101.4	83.8			
	*	Burkina Faso	208.4	148.8	97.3	128.9	107.9			
	*	Burundi	182.6	141.7	85.2	128.6	100.1			
	*	Cambodia	116.7	46.0	54.5	58.8	41.9	✓	✓	
	*		169.1	164.6	78.9	130.6	99.6			
	*	Chad	208.3	171.3	97.2	143.1	106.7			
	*	Comoros	121.7	81.3	56.8	76 · 5	58.4			
	*	Congo, Dem. Rep.	181.4	169.9	84.7	153.1	115.2			
	*	Eritrea	137.7	69.8	64.3	71.4	55.6		✓	
	*	Ethiopia	198.3	81.5	92 · 5	95.1	74.5	✓	✓	
	*	Gambia, The	164.6	102.6	76.8	96.8	79.2			
	*	Guinea	228.2	129.8	106.5	128.5	101.3			
	*	Guinea-Bissau	210.4	161.7	98.2	141.7	110.6			
	*	Haiti	143.0	160.7	66.7	80.4	59.9			
	*	Kenya	97.8	76.1	45.6	75.7	60.0			
	*	Kyrgyz Republic	70.3	31.7	32.8	35.2	29.0	✓	✓	
	*	Liberia	241.2	83.1	112.6	105.1	83.8	✓	✓	✓
	*	Madagascar	161.2	64.3	75.2	77.5	59.4	✓	✓	
	*	Malawi	227.0	89.0	105.9	110.6	88.2	✓	✓	
**LOW INCOME GROUP**	*	Mali	257.3	178.9	120.1	157.0	127.0			
	*	Mozambique	225.7	108.4	105.3	116.2	93.2		✓	
	*	Nepal	134.6	50 .3	62.8	58.2	47.6	✓	✓	
	*	Niger	313.7	130.9	146.4	159.8	131.6	✓	✓	✓
	*	Rwanda	156.3	60.4	72.9	104.3	84.3	✓	✓	✓
	*	Sierra Leone	266.7	188.8	124.5	179.8	141.1			
	*	Tajikistan	114.3	66.1	53.3	74.6	63.6		✓	
	*	Tanzania	157.9	72.5	73.7	85.7	71.4	✓	✓	
	*	Togo	147.0	111.7	68.6	96.0	76.5			
	*	Uganda	178.0	94.2	83.1	94.8	74.4		✓	
	*	Zimbabwe	79.2	72.4	37.0	67.9	49.0			
**LOW-MIDDLE INCOME GROUP**		Armenia	47.2	18.3	22.0	21.3	17.3	✓	✓	
		Bhutan	138.4	55.9	64.6	58.9	50.7	✓	✓	
	*	Bolivia	119.5	52.9	55.8	57.4	42.0	✓	✓	
	*	Cameroon	145.2	128.9	67.8	105.7	81.1			
		Cape Verde	58.0	22.5	27.1	25.4	21.3	✓	✓	
	*	Congo, Rep.	118.8	99.6	55.4	82.6	65.9			
	*	Cote d'Ivoire	151.4	116.7	70.7	104.0	82.5			
	*	Djibouti	121.6	91.2	56.7	82.8	60.1			
	*	Egypt, Arab Rep.	85.7	22.5	40.0	30.7	25.2	✓	✓	✓
		El Salvador	60.2	16.3	28.1	22.8	19.2	✓	✓	✓
		Georgia	46.9	21.5	21.9	25.4	19.6	✓	✓	
	*	Ghana	120.9	79.6	56.4	70.1	55.1			
	*	Guatemala	78.0	31.6	36.4	34.9	29.4	✓	✓	
		Guyana	63.0	37.0	29.4	33.6	25.7	✓	✓	
		Honduras	55.0	22.2	25.7	25.6	22.1	✓	✓	
	*	India	114.2	63.4	53.3	59.2	48.7			
	*	Indonesia	81.6	33.3	38.1	36.4	26.2	✓	✓	
		Kiribati	87.6	48.7	40.9	47.2	41.8			
	*	Lao PDR	147.7	43.9	68.9	54.5	36.7	✓	✓	
	*	Lesotho	87.5	93.0	40.8	74.2	65.9			
	*	Mauritania	124.7	112.9	58.2	88.5	69.8			
		Micronesia, Fed. Sts.	56.4	42.0	26.3	35.8	31.5		✓	
		Moldova	34.9	16.6	16.3	18.9	15.1	✓	✓	
		Mongolia	106.5	33.0	49.7	44.1	38.7	✓	✓	✓
	*	Morocco	81.3	34.3	37.9	37.9	31.0	✓	✓	
		Nicaragua	66.1	27.0	30.8	30.3	25.5	✓	✓	
**LOW-MIDDLE INCOME GROUP**	*	Nigeria	213.6	129.2	99.7	129.2	100.7		✓	
	*	Pakistan	122.2	73.7	57.0	69.3	55.4			
	*	Papua New Guinea	88.0	59.5	41.1	52.8	44.9			
		Paraguay	52.6	23.4	24.5	26.0	18.1	✓	✓	
	*	Philippines	57.0	26.4	26.6	28.1	19.7	✓	✓	
		Samoa	29.5	18.9	13.8	16.7	15.0	✓	✓	
	*	Senegal	135.9	69.1	63.4	83.8	68.9		✓	
	*	Solomon Islands	41.8	22.2	19.5	22.8	19.7	✓	✓	
		Sri Lanka	28.9	12.6	13.5	13.0	10.9	✓	✓	
	*	Sudan	122.8	87.7	57.3	73.5	59.4			
	*	Swaziland	83.3	109.2	38.9	76.3	67.7			
		Syrian Arab Republic	36.1	15.9	16.8	16.5	12.6	✓	✓	
		Timor-Leste	180.0	57.6	84.0	63.5	45.5	✓	✓	
		Ukraine	19.4	10.7	9.1	13.4	11.1	✓	✓	✓
	*	Uzbekistan	75.3	49.6	35.1	46.5	38.2			
		Vanuatu	38.5	13.7	18.0	16.5	14.7	✓	✓	✓
	*	Vietnam	49.9	22.6	23.3	22.4	16.3	✓		
		West Bank and Gaza	43.1	22.6	20.1	22.0	16.7	✓	✓	
	*	Yemen, Rep.	126.0	78.5	58.8	72.9	50.4			
	*	Zambia	192.8	90.4	90.0	110.6	86.5	✓	✓	
**UPPER-MIDDLE INCOME GROUP**		Albania	41.2	15.0	19.2	17.7	15.4	✓	✓	✓
		Algeria	65.6	31.3	30.6	33.6	28.3	✓	✓	
	*	Angola	243.2	161.0	113.5	146.9	116.0			
		Argentina	27.6	14.5	12.9	14.1	11.7	✓	✓	
	*	Azerbaijan	94.5	46.4	44.1	51.0	37.8		✓	
		Belarus	17.2	6.1	8.0	8.4	7.7	✓	✓	✓
		Belize	43.9	17.6	20.5	19.0	15.8	✓	✓	
		Bosnia and Herzegovina	18.8	7.9	8.8	6.0	5.4	✓	✓	
	*	Botswana	52.8	27.5	24.6	37.7	35.6	✓	✓	✓
	*	Brazil	58.0	16.8	27.1	24.2	19.6	✓	✓	✓
		Bulgaria	22.2	12.7	10.4	14.3	12.9	✓	✓	✓
	*	China	48.9	15.9	22.8	19.7	17.9	✓	✓	✓
		Colombia	34.3	18.3	16.0	18.1	13.9	✓	✓	
		Costa Rica	17.2	10.1	8.0	9.1	7.6	✓	✓	
		Cuba	13.3	5.9	6.2	6.3	5.3	✓	✓	
		Dominica	17.4	12.1	8.1	10.6	9.4	✓	✓	
		Dominican Republic	58.3	25.7	27.2	26.4	21.4	✓	✓	
		Ecuador	52.4	23.6	24.5	25.4	19.5	✓	✓	
		Fiji	29.6	17.1	13.8	16.3	15.1	✓	✓	
	*	Gabon	94.4	67.4	44.1	62.0	46.4			
		Grenada	21.0	13.1	9.8	11.4	9.7	✓	✓	
		Hungary	18.7	6.6	8.7	7.6	6.8	✓	✓	✓
		Iran, Islamic Rep.	61.1	26.2	28.5	29.2	24.4	✓	✓	
	*	Iraq	46.0	38.6	21.5	30.5	22.0	✓		
		Jamaica	34.5	19.0	16.1	18.6	15.4	✓	✓	
		Jordan	36.7	21.1	17.1	20.3	15.9	✓	✓	
		Kazakhstan	57.0	29.2	26.6	31.1	27.1	✓	✓	
		Lebanon	33.1	9.9	15.4	12.4	10.2	✓	✓	✓
		Libya	44.1	17.0	20.6	18.3	16.0	✓	✓	
		Macedonia, FYR	37.6	10.2	17.5	13.1	11.2	✓	✓	✓
		Malaysia	17.2	6.8	8.0	7.3	5.2	✓	✓	
		Maldives	105.2	12.4	49.1	24.6	20.5	✓	✓	✓
		Marshall Islands	51.9	27.7	24.2	28.0	25.7	✓	✓	
		Mauritius	23.9	15.2	11.2	12.8	10.5	✓	✓	
	*	Mexico	48.8	16.6	22.8	20.6	17.7	✓	✓	✓
		Montenegro	17.6	7.6	8.2	8.4	7.2	✓	✓	
		Namibia	72.8	45.9	34.0	48.2	42.4		✓	
		Palau	32.3	19.1	15.1	18.9	17.5	✓	✓	
		Panama	33.3	20.0	15.5	17.9	15.3	✓	✓	
	*	Peru	75.1	19.4	35.0	26.9	20.9	✓	✓	✓
		Romania	37.4	13.6	17.5	18.2	15.1	✓	✓	✓
		Serbia	28.6	7.4	13.3	10.4	8.5	✓	✓	✓
		Seychelles	16.6	13.9	7.7	10.0	8.1	✓	✓	
	*	South Africa	62.3	52.6	29.1	50.2	44.1			
		St. Lucia	22.5	15.8	10.5	13.1	10.8	✓	✓	
		St. Vincent & the Grenadines	26.5	21.2	12.4	16.0	14.2	✓	✓	
		Suriname	51.9	29.8	24.2	29.5	23.1	✓	✓	
		Thailand	35.0	12.8	16.3	13.4	9.4	✓	✓	
		Tonga	24.5	15.7	11.4	14.1	13.2	✓	✓	
		Tunisia	51.1	17.2	23.8	20.5	16.9	✓	✓	
		Turkey	72.0	16.3	33.6	24.3	19.7	✓	✓	✓
	*	Turkmenistan	94.3	54.0	44.0	51.7	43.5			
		Tuvalu	57.6	31.1	26.9	30.5	28.1	✓	✓	
		Venezuela, RB	30.9	15.6	14.4	16.2	12.6	✓	✓	
HIGH INCOME GROUP**		Chile	18.7	8.8	8.7	7.9	6.4	✓	✓	
		Croatia	12.9	5.3	6.0	5.7	4.9	✓	✓	
		Czech Republic	14.3	4.1	6.7	4.8	4.3	✓	✓	✓
	*	Equatorial Guinea	189.6	122.0	88.5	84.9	73.8			
		Estonia	20.1	4.1	9.4	6.1	5.8	✓	✓	✓
		Latvia	20.6	8.9	9.6	11.6	11.6	✓	✓	✓
		Lithuania	17.4	6.2	8.1	8.4	8.1	✓	✓	✓
		Oman	47.5	9.4	22.2	15.0	11.4	✓	✓	✓
		Poland	17.3	6.1	8.1	6.5	5.9	✓	✓	
		Russian Federation	27.3	12.5	12.7	15.1	12.5	✓	✓	✓
		Slovak Republic	17.6	8.0	8.2	8.5	7.7	✓	✓	
		Trinidad and Tobago	36.8	28.0	17.2	22.9	18.6	✓	✓	
		Uruguay	23.1	10.8	10.8	11.6	9.8	✓	✓	
**Percent of countries on track:**								**69%**	**74%**	**20%**
**Percent of SSA countries on track:**								**27%**	**40%**	**9%**

*Countdown Country. Six out of the 75 countdown^5^ countries are not included in this list & analysis because these where missing GDP per capita data: Afghanistan, Korea DPR, Myanmar, Sao Tome & Principe, Somalia, and South Sudan.

**Note that these high income countries were not classified as high income in 2000, which was the cut-off point for this analysis. The list is based on the income in 2013. † ‘On track’ indicates that the under-five mortality rate is less than 40 deaths per 1,000 live births or that it is 40 or more with an average annual rate of reduction of 4% or higher for 1990–2010.^7^

According to our fast-track U5MR target, only 29 countries out of 144 (20%) are on the fast track. Of these top performers, 4 are in Africa (Botswana, Liberia, Niger and Rwanda). On average across countries the minimum performance target requires achieving a U5MR in 2010 that is 25% higher than the MDG 4 target for 2010. The fast-track target on average requires a U5MR that is 5% lower in 2010 than the MDG target, although in SSA the average fast-track target is actually 5% higher (i.e. less ambitious) than the average MDG target.

Likewise, Table 4 shows the original starting MMR of each country in our dataset in 1990, the latest estimate for 2010, and what a country’s MMR would have been in 2010 if it were on a linear path to achieve a 75% reduction in the 1990 MMR by 2015. Of 116 countries available for analysis, 25 (22%) are on track by the MDG 5 target. Of the 42 sub Saharan African countries studied, 5 (12%) are on track by the MDG 5 target. In contrast, under the MMR minimum performance target, 69 countries of 116 (59%) would be considered on track and 25 of 42 (60%) sub Saharan African countries would be considered on track. On the other hand, for the MMR fast-track target, eight countries of 116 (7%) would be considered on track and 0 of 42 (0%) of sub-Saharan African countries would be considered on track (Figure 2C-D). On average, the minimum performance target requires an MMR for 2010 that is 67% higher than the MDG target would require. The *fast-track target* requires a 2010 MMR that is 30% lower than the MDG target for 2010.

**Table 4:** **MDG 5a: Countries’ performance based on global, minimum performance and fast-track targets listed by income group**

			**MMR MDG target**			**MMR alternative 1 minimum performance target**	**MMR alternative 2 fast-track target**	**SUMMARY OF COUNTRIES ON TRACK†**		
			**(Time projections only)**			**(Time & GDP adjusted)**	**(Time, GDP, & X-factor adjusted)**			
Income group		Country	MMR Actual 1990	MMR Actual 2010	*MDG 5a Target for 2010*	*2010 Target adjusted for GDP and country trajectory*	*2010 Target based on adoption of best attainable policies in the region*	On track by global MDG 5a target	On track by minimum performance target	On track by fast-track target
**LOW INCOME GROUP**	*	Bangladesh	800.0	240.0	320.0	392.7	216.4	✓	✓	
	*	Benin	770.0	350.0	308.0	488.0	166.8		✓	
		Burkina Faso	700.0	300.0	280.0	379.2	122.9		✓	
		Burundi	1100.0	800.0	440.0	1137.4	308.3		✓	
	*	Cambodia	830.0	250.0	332.0	346.4	207.8	✓	✓	
	*	Central African Republic	930.0	890.0	372.0	820.8	411.0			
		Chad	920.0	1100.0	368.0	635.3	298.4			
		Comoros	440.0	280.0	176.0	391.0	160.9		✓	
		Congo, Dem. Rep.	930.0	540.0	372.0	1667.5	504.7		✓	
	*	Eritrea	880.0	240.0	352.0	450.1	97.0	✓	✓	
	*	Ethiopia	950.0	350.0	380.0	604.3	173.2	✓	✓	
	*	Gambia, The	700.0	360.0	280.0	512.2	198.3		✓	
		Guinea	1200.0	610.0	480.0	873.0	298.6		✓	
	*	Guinea-Bissau	1100.0	790.0	440.0	921.8	267.8		✓	
		Haiti	620.0	350.0	248.0	490.4	78.1		✓	
		Kenya	400.0	360.0	160.0	319.2	115.4			
	*	Kyrgyz Republic	73.0	71.0	29.2	72.0	45.6		✓	
	*	Liberia	1200.0	770.0	480.0	791.2	282.4		✓	
	*	Madagascar	640.0	240.0	256.0	566.2	154.7	✓	✓	
		Malawi	1100.0	460.0	440.0	661.1	169.0		✓	
		Mali	1100.0	540.0	440.0	681.0	241.5		✓	
	*	Mozambique	910.0	490.0	364.0	453.0	182.5			
	*	Nepal	770.0	170.0	308.0	424.1	160.3	✓	✓	
		Niger	1200.0	590.0	480.0	1062.5	282.5		✓	
		Rwanda	910.0	340.0	364.0	593.1	197.5	✓	✓	
		Sierra Leone	1300.0	890.0	520.0	1191.9	419.9		✓	
		Tajikistan	94.0	65.0	37.6	147.5	70.0		✓	✓
		Tanzania	870.0	460.0	348.0	560.9	211.8		✓	
		Togo	620.0	300.0	248.0	493.6	143.6		✓	
		Uganda	600.0	310.0	240.0	282.5	126.6			
		Zimbabwe	450.0	570.0	180.0	381.9	137.1			
**LOW-MIDDLE INCOME GROUP**	*	Armenia	46.0	30.0	18.4	32.3	22.6		✓	
		Bhutan	1000.0	180.0	400.0	371.1	132.6	✓	✓	
	*	Bolivia	450.0	190.0	180.0	269.7	109.8		✓	
		Cameroon	670.0	690.0	268.0	567.1	295.2			
		Cape Verde	200.0	79.0	80.0	83.0	36.7	✓	✓	
	*	Congo, Rep.	420.0	560.0	168.0	341.7	228.2			
		Cote d'Ivoire	710.0	400.0	284.0	602.8	202.8		✓	
		Djibouti	290.0	200.0	116.0	332.8	102.4		✓	
		Egypt, Arab Rep.	230.0	66.0	92.0	121.7	89.8	✓	✓	✓
	*	El Salvador	150.0	81.0	60.0	75.2	48.7			
		Georgia	63.0	67.0	25.2	93.7	37.9		✓	
		Ghana	580.0	350.0	232.0	322.2	110.5			
		Guatemala	160.0	120.0	64.0	98.3	50.6			
		Guyana	180.0	280.0	72.0	78.2	49.9			
		Honduras	220.0	100.0	88.0	144.1	64.3		✓	
		India	600.0	200.0	240.0	255.0	136.2	✓	✓	
		Indonesia	600.0	220.0	240.0	290.8	277.6	✓	✓	✓
		Lao PDR	1600.0	470.0	640.0	680.4	308.5	✓	✓	
	*	Lesotho	520.0	620.0	208.0	289.0	166.3			
	*	Mauritania	760.0	510.0	304.0	536.6	191.1		✓	
		Micronesia, Fed. Sts.	140.0	100.0	56.0	91.5	88.4			
	*	Mongolia	120.0	63.0	48.0	82.4	44.9		✓	
		Morocco	300.0	100.0	120.0	181.3	96.7	✓	✓	
		Nicaragua	170.0	95.0	68.0	107.2	50.2		✓	
		Nigeria	1100.0	630.0	440.0	688.8	293.2		✓	
	*	Pakistan	490.0	260.0	196.0	296.1	105.7		✓	
		Papua New Guinea	390.0	230.0	156.0	241.6	163.3		✓	
	*	Paraguay	120.0	99.0	48.0	89.0	39.8			
		Philippines	170.0	99.0	68.0	110.3	84.0		✓	
		Samoa	260.0	100.0	104.0	156.9	111.8	✓	✓	✓
		Senegal	670.0	370.0	268.0	477.5	140.5		✓	
	*	Solomon Islands	150.0	93.0	60.0	111.4	49.1		✓	
		Sri Lanka	85.0	35.0	34.0	36.0	19.4		✓	
		Sudan	1000.0	730.0	400.0	489.7	581.0			
	*	Swaziland	300.0	320.0	120.0	177.3	107.0			
		Syrian Arab Republic	240.0	70.0	96.0	129.2	44.4	✓	✓	
	*	Timor-Leste	1000.0	300.0	400.0	455.2	231.4	✓	✓	
		Uzbekistan	59.0	28.0	23.6	24.3	17.4			
		Vanuatu	220.0	110.0	88.0	144.8	90.6		✓	
	*	Vietnam	240.0	59.0	96.0	81.6	52.7	✓	✓	
	*	Yemen, Rep.	610.0	200.0	244.0	395.3	55.7	✓	✓	
	*	Zambia	470.0	440.0	188.0	394.2	147.8			
**UPPER-MIDDLE INCOME GROUP**	*	Algeria	220.0	97.0	88.0	159.3	74.0		✓	
	*	Argentina	71.0	77.0	28.4	35.7	26.8			
		Azerbaijan	56.0	43.0	22.4	50.7	28.7		✓	
	*	Belize	71.0	53.0	28.4	38.0	17.7			
	*	Botswana	140.0	160.0	56.0	68.3	40.0			
	*	Brazil	120.0	56.0	48.0	77.0	39.5		✓	
	*	China	120.0	37.0	48.0	25.9	51.8	✓		✓
	*	Colombia	170.0	92.0	68.0	106.4	73.9		✓	
	*	Costa Rica	38.0	40.0	15.2	19.9	11.5			
	*	Cuba	63.0	73.0	25.2	49.4	34.0			
	*	Dominican Republic	220.0	150.0	88.0	97.8	69.5			
	*	Ecuador	180.0	110.0	72.0	122.3	56.9		✓	
	*	Fiji	32.0	26.0	12.8	20.5	13.4			
	*	Gabon	270.0	230.0	108.0	225.9	163.3			
	*	Grenada	34.0	24.0	13.6	18.3	8.7			
	*	Iran, Islamic Rep.	120.0	21.0	48.0	63.5	17.5	✓	✓	
	*	Iraq	89.0	63.0	35.6	59.0	16.4			
	*	Jamaica	59.0	110.0	23.6	38.4	27.6			
	*	Jordan	110.0	63.0	44.0	67.7	18.5		✓	
	*	Kazakhstan	92.0	51.0	36.8	54.5	77.1		✓	✓
	*	Lebanon	52.0	25.0	20.8	20.7	18.2			
	*	Libya	99.0	58.0	39.6	51.8	42.1			
	*	Malaysia	53.0	29.0	21.2	22.8	19.3			
	*	Maldives	830.0	60.0	332.0	174.9	91.7	✓	✓	✓
		Mauritius	68.0	60.0	27.2	29.9	23.0			
	*	Mexico	92.0	50.0	36.8	56.3	36.1		✓	
		Namibia	200.0	200.0	80.0	122.5	72.2			
	*	Panama	100.0	92.0	40.0	46.3	47.8			
	*	Peru	200.0	67.0	80.0	105.8	61.6	✓	✓	
	*	South Africa	250.0	300.0	100.0	185.0	117.8			
	*	St. Lucia	64.0	35.0	25.6	36.8	17.7		✓	
	*		59.0	48.0	23.6		19.2			
	*	Suriname	84.0	130.0	33.6	60.6	35.1			
	*	Thailand	54.0	48.0	21.6	25.1	17.3			
	*	Tonga	67.0	110.0	26.8	37.9	46.6			
	*	Tunisia	130.0	56.0	52.0	64.1	39.3		✓	
	*	Turkey	67.0	20.0	26.8	36.4	26.7	✓	✓	✓
	*	Turkmenistan	82.0	67.0	32.8	55.7	48.7			
	*	Venezuela, RB	94.0	92.0	37.6	66.0	39.3			
HIGH INCOME GROUP**	*	Chile	56.0	25.0	22.4	23.5	14.4			
		Oman	110.0	32.0	44.0	58.8	12.2	✓	✓	
	*	Trinidad and Tobago	86.0	46.0	34.4	42.9	35.2			
	*	Uruguay	39.0	29.0	15.6	20.0	13.2			
**Percent of countries on track:**								**22%**	**59%**	
**Percent of SSA countries on track:**								**12%**	**60%**	**0%**

*Countdown Country. Eight out of the 75 countdown^5^ countries are not included in this list and analysis because these countries had missing data for GDP per capita: Afghanistan, Angola, Equatorial Guinea, Korea DPR, Myanmar, Sao Tome & Principe, Somalia, South Sudan. Two others were excluded because there were zero nationally reported figures from 1990–2011: Angola, Equatorial Guinea **Note that these high income countries were not classified as high income in 2000, which was the cut-off point for this analysis. The list is based on the income in 2013.

† ‘On track’ indicates that absolute maternal mortality ratio by 2010 has reduced to 60%.^7^

## Discussion

If one interprets the intent of the Millennium Declaration as an aspiration for the entire human population, the world as a whole is on track to achieve MDG 4 by 2015. As of 2010, the population weighted average reduction in U5MR since 1990 was 55 percent (Table 2). Extrapolating past performance, where U5MR drops 2.75 (=55/20) percentage points per year for five more years towards 2015 yields the projection that the world will have attained a 68.75% (=55 + (2.75 × 5)) reduction in U5MR by 2015—meeting the 2015 goal. However, if weighted by number of births, rather than population, progress would be less. The 144 LMIC countries in our sample as a whole have only reduced a birth-cohort-weighted U5MR by 39.5%, because large countries that maintained high levels of fertility rate, i.e. D.R. Congo, Nigeria, and India have made less overall progress than the large countries that reduced their birth cohort significantly: Bangladesh, Indonesia, Brazil, and China. Reasons to prefer population weighted vs. birth cohort weighted estimates are discussed in the Additional file 1: Appendix.

These findings demonstrate the value of global measurements as a simple and intelligible method to evaluate global progress and strengthen aspirations for further improvements. However, if one pursues a separate objective of assessing individual country performance, then applying a single benchmark to all countries can be inappropriately alarmist. Our analysis showed that country-specific targets based on each country’s specific trajectory of GDP growth enables more countries to receive the credit they deserve for their progress. In particular the *minimum performance targets* (Table 3) revealed that in Africa, Eritrea, Mozambique, Namibia, Nigeria, Senegal, and Uganda have been making an acceptable degree of progress given their resource constraints. Conventional MDG-based standards show these six countries to be not on track, which is overly pessimistic. Poorer than average GDP growth and unfortunate starting trajectories in these countries were critical contributors to their failure to obtain a 66% reduction in child mortality.

The HIV epidemic in Africa made achieving the global MDGs there significantly more difficult. The fact that only 27% of sub-Saharan African countries are on track for MDG4 and 12% are on track for MDG5a is in no doubt partly due to HIV, in addition to the other challenges faced by these countries. Indeed, the MDG4 target for under-5 mortality was even more ambitious than the fast-track target in this analysis, underscoring how ambitious—and unrealistic—such a goal was for most countries in the region. That the HIV epidemic did not factor at all in setting the MDG mortality targets in Africa demonstrates the limitations of applying global goals to each country in a diverse world. Both our targets target allows a more nuanced evaluation of African countries’ progress.

In addition to disclosing that progress has been higher than anticipated, the minimum target makes the detection of stalled progress more salient. Using a GDP-adjusted performance target to detect countries that are off track on maternal and child health implies that their stalled progress cannot be simply due to low national income. Failing to do as well as comparable countries have, controlling for GDP, implies a need for more accountability and better stewardship of a country’s resources. For suggestions about how to achieve exceptional results one can turn to the fast-track countries.

The *fast-track targets* were based on what would have happened to U5MR and MMR had a country experienced the regionally best possible improvements in ten known determinants of child survival. These more stringent targets highlight the success in lowering U5MR of Botswana, Liberia, Niger, and Rwanda in sub-Saharan Africa. Other top performers are Brazil, China, Egypt, El Salvador, Lebanon, Maldives, Mexico, Mongolia, Oman, Peru, Turkey, Vanuatu, and most LMIC European countries. Case studies of these high performers are proving their value in showing how these countries are achieving results beyond what would be expected [47-51].

Our study revealed that many African countries are actually meeting the minimum performance target and do not deserve to be unfairly labeled as “off track”. Many so called “off track” countries have actually achieved what was reasonably achievable given their available resources. Yet still, more than half of sub-Saharan Africa has not met a minimum standard, even adjusting for their economic circumstances. Failing to meet the minimum performance target suggests that the chief problem is not low GDP growth, but below-average capacity to transform economic growth into better health. The remedy for countries that are not achieving GDP adjusted targets will not lie in economic growth, but rather in better use of existing resources to improve health. Examining success factors for the superior performers on the *fast-track* standard for the region can indicate how countries have achieved better value for money. Botswana, Liberia, Niger, and Rwanda are distinguished in this category. Botswana has a relatively high GDP, but some of its success can be attributed to having the best control of corruption in all of sub-Saharan Africa and the second best access to clean water (after Mauritius). Botswana also has nearly complete vaccine coverage. Rwanda’s superb performance in maternal and child health has been well documented, especially lower financial barriers for health services [47] and dramatic improvements in coverage for rural attended deliveries [48]. Rwanda also had the fourth best control of corruption score in sub-Saharan Africa in 2010. Niger started out with the worst U5MR in the world in 1990, and has now improved to about average in its sub-region, by improving to average its control of corruption, measles immunization rates, and female primary schooling, despite still performing poorly on the other indicators in the model [51]. Liberia’s recent successes and challenges were detailed in a case study [52].

Four countries—China, Egypt, Maldives, and Turkey—all MICs, meet the *fast-track target* for both MDG4 and 5. China’s success is well known, [53] and we predict that case studies of Egypt, Turkey and Maldives public health policies will be particularly instructive.

### Limitations

This study is limited by some uncertainty in the measurement of U5MR and MMR given that many countries that lack vital registration systems and the available data are based on modelled estimates. The statistical model used in this analysis is more complex than a single global target that can perhaps be more easily understood by the general public. The need for statistical techniques to develop country-specific targets may reduce political intelligibility. Additionally, the minimum performance target could lead some countries to be unnecessarily restrained in their ambition, and careful framing would be necessary to avoid this. It is important to note that this study does not attempt to provide new evidence on the factors or set of factors that most impact child/maternal health.

### Policy implications

Contrary to a common prevalent discourse that there has been a pervasive failure to meet MDG-based expectations for progress in in maternal and child health, our analysis implies a less dire and more nuanced situation. The remarkable outpouring of attention to reducing preventable maternal and child deaths has revealed many success stories in places that had been overlooked before.

The recent history of how the MDGs were used as country performance targets should alert policy makers that any global goals announced for the post-2015 era will be similarly treated. It is also a forewarning of the urgent need for the development community to apply targets that correctly flag both high performers and low performers. This paper demonstrates two illustrative prototypes for the creation of evidence-informed, country-specific targets that can help take forward the post-2015 agenda. Our approach uses primarily a best statistical fit model within a set of known success factors, but stakeholders could consider alternative local adjustments, such as the best observed rate of progress of another country in their region or income group [9].

While global goals have clear value for inducing shared commitments and simplicity for a broad audience, targets that are informed by specific country situations can supplement global targets. Global targets do not take account of special circumstances in each country. National policymakers typically know their special circumstances and justify making allowances if they are not meeting global targets. The absence of appropriate country-specific targets can actually impede the ability of civil society to hold national governments accountable for achievable progress. When countries correctly identify that they have not made progress relative to their own potential, it can help organize their own efforts. Having an additional set of customized performance measures would help local decision-makers monitor their own progress rather than deferring to global experts and global criteria. Recent scholarship has documented several cases where policy domination by outside experts has inhibited the growth of accountable local systems of governance [6]. An approach that builds on our demonstration would involve additional stakeholders in developing politically intelligible, country-specific targets to help national policy makers and stakeholders accurately assess their performance relative to their potential. Table 5 displays several prominent options for country-specific targets, and shows that there are a variety of options that can be used to supplement global goals when assessing country performance in mortality reduction.

**Table 5:** **Different country-specific targets available for policy-makers to consider**

1)	Global targets
2)	GDP-adjusted minimum performance targets
3)	‘Success-factor’ adjusted fast-track targets [10,54]
4)	Regional best performance targets [9]
5)	Targets based on assumptions of new technologies and improved performance [55]

## Additional file

Additional file 1
**Appendix.**
