# Supplementary material for: Country progress towards the Millennium Development Goals: adjusting for socioeconomic factors reveals greater progress and new challenges
Source: Global Health. 2014 Oct 1;10:67. doi: 10.1186/s12992-014-0067-7 (PMC4197219; doi:10.1186/s12992-014-0067-7)
Supplement: Additional file 1 — Appendix. [file 12992_2014_67_MOESM1_ESM.docx]

**Country progress towards Millennium Development Goals: Adjusting for socioeconomic factors reveals greater progress and new challenges**

**APPENDIX**

TABLE OF CONTENTS

[INTRODUCTION 2](#_Toc381702392)

[SECTION 1 - METHODS 2](#_Toc381702393)

[DATA SOURCES 2](#_Toc381702394)

[COUNTRY SELECTION 2](#_Toc381702395)

[Table I. Countries and sub-regions used to form the fast-track target 3](#_Toc381702396)

[VARIABLES SELECTION 4](#_Toc381702397)

[Table IIa. Variables considered for inclusion in developing the fast-track target 6](#_Toc381702398)

[Table IIb. Variables included in U5MR and MMR models by priority area 14](#_Toc381702399)

[METHODS 14](#_Toc381702400)

[Overall Procedure 14](#_Toc381702401)

[Statistical Model 14](#_Toc381702402)

[Regression Equation 15](#_Toc381702403)

[Building the Targets 16](#_Toc381702404)

[Table IIIa. Variables and regressions test for the U5MR model 18](#_Toc381702405)

[Table IIIb. Variables and regressions test for the MMR model 19](#_Toc381702406)

[SECTION 2 - RESULTS 20](#_Toc381702407)

[Table IVa. Regression results - U5MR targets 21](#_Toc381702408)

[Table IVb. Regression results - MMR targets 24](#_Toc381702409)

[SECTION 3 - UNCERTAINTY ANALYSIS 26](#_Toc381702412)

[Imputation 26](#_Toc381702413)

[Weighting to Make a Global Aggregate 26](#_Toc381702414)

[Uncertainty Range 26](#_Toc381702415)

[Table V. Uncertainty analysis ranges 27](#_Toc381702416)

[BIBLIOGRAPHY 33](#_Toc381702417)

# INTRODUCTION

This appendix has 3 sections. Section 1 describes data sources, selection of countries into the sample, how variables were selected and statistical methods.  Section 2 describes the statistical results from regression analysis and results by country and world region. Section 3 describes uncertainty analysis and uncertainty bounds around the country targets.

# SECTION 1 - METHODS

## DATA SOURCES

Data for outcome and independent variables of interest was extracted and compared between the following sources: WHO Global Health Observatory[[1](#_ENREF_1)], WHO National Health Accounts (NHA),[[2](#_ENREF_2)] UNdata,[[3](#_ENREF_3)] UNDP,[[4](#_ENREF_4)] UNICEF Childinfo,[[5](#_ENREF_5)] the World Bank DataBank,[[6](#_ENREF_6)] and Demographic and Health Surveys (DHS).[[7](#_ENREF_7)] Completeness of the dataset varied by indicator, see Table 1. For MMR data, both modeled and national estimates were available. Our model uses the national estimates for which the original source is UNICEF’s Childinfo and DHS. For U5MR, World Bank estimates are developed by the UN Inter-agency Group for Child Mortality Estimation (IGME) (UNICEF, WHO, World Bank, UN DESA, UNDP). For those independent variables lacking complete data, simple regression imputation was used to fill in missing data, using ordinary least squares regression (OLS) to predict the missing independent variables against log GDP per capita, year, UN subregion, urbanization, total fertility rate (TFR), and ethnic fractionalization.[[8](#_ENREF_8)] The effect of imputing was to allow the production of targets for countries that would have been left out otherwise. Robustness checks (detailed below) indicate that inclusion of imputed data did not appreciably change the final overall targets calculated.

## COUNTRY SELECTION

Analysis was confined to the 193 UN member states plus the Occupied Palestinian Territories. Countries were excluded from analysis if they had no reported values for GDP, under-5 mortality, or maternal mortality from 1990-2011. We excluded all countries classified as high-income by their 2000 GDP per capita (defined as $9,266[[9](#_ENREF_9)]  in 2000 US dollars) from both analyses. We additionally excluded all European countries from the MMR analysis, since MMR in those countries is comparable to high-income countries. These criteria left 144 countries eligible for the MDG4 analysis and 116 countries in the MDG5 analysis. The list of countries included the final analysis is in the appendix Table I. Countries were grouped in sub-regions as defined by the UN statistics division.[[10](#_ENREF_10)]

### Table I. Countries and sub-regions used to form the fast-track target

## VARIABLES SELECTION

**Dependent Variables**

The dependent variables used in the models are under-5 mortality rate (U5MR) and maternal mortality ratio (MMR). Given that most recent MDG summits and reports call to mobilize and intensify global action to improve the health of women and children around the world due to the insufficient progress made in MDGs 4 and 5, our analysis focuses on these two global health goals.[[11-13](#_ENREF_11)] U5MR is the probability per 1,000 live births that a newborn baby will die before reaching age five, if subjected to current age-specific mortality rates, and MMR is the number of women who die from causes related to or aggravated by pregnancy and its management during pregnancy and childbirth or within 42 days of pregnancy per 100,000 live births.[[6](#_ENREF_6)]

A limitation of using UN interagency data on U5MR and MMR is that the official mortality statistics of many countries cannot be considered random variables. Many are calculated variables using splines with an error structure that does not conform to the assumptions of a regression model. This limitation would invalidate an attempt to rely on regression to prove that a statistical association between mortality and factor “X” is significant according to a P-value. This paper therefore does not aspire to prove assertions about the determinants of mortality. The goal is to set targets that are relevant to monitoring country-specific progress. The regression coefficients we use to set targets remain reliable (although the standard errors around these coefficients are not) and these coefficients will be used to produce the predictions required to set targets. The analysis is careful to only use independent variables whose statistical significance in predicting mortality has already been proven in past studies. However, despite the error structure of UN data, the potential alternative estimates from the Institute of Health Metrics and Evaluation are also constructed in part using models and thus bear a similar problem. Furthermore, IGME and IHME estimates are highly correlated, with a correlation coefficient greater than 0.98.

**Independent Variables**

Since progress towards reducing child and maternal mortality is influenced by multifactorial variables and is at least somewhat related to economic development, a broad perspective on health and societal development was used to select independent variables to include in models. Firstly, the UN suggests that plans designed to meet the MDGs should include investments in seven “clusters” of public policy.[[14](#_ENREF_14)] We use these clusters as a guide to group variables into priority areas that could represent a complete set of resources and health and socioeconomic public practices that impact child/maternal health.

To try to make the priority areas mutually exclusive we adjust the UN’s clusters into a total of 10 priority areas. These are, in brief: 1) education, 2) nutrition, 3) environmental management, 4) infrastructure, 5) population dynamics, 6) inequality divided into 6a) socioeconomic inequality and 6b) gender inequality, 7) governance, 8) economic growth, 9) burden of disease, and 10) health systems. Health systems is broken-down according to the six building blocks as defined by the WHO[[15](#_ENREF_15)]: 1) financing, 2) policies, 3) Information systems, 4) human resources, 5) service coverage/delivery and 6) technologies. A list of indicators that could impact child or maternal health gathered from the data sources listed previously were reviewed by child and maternal health experts from the World Health Organization. Over 250 candidate independent variables were considered and grouped by priority area.^7^ For a list of variables see appendix Table IIa.

We attempt to include at least one variable from each priority area and subcategories because we found that there was significant collinearity between related factors, and because it was important to avoid over-specificity in the model. [[16-21](#_ENREF_16)] However, we consider it inappropriate to include variables from the priority areas nutrition and burden of disease since that would be regressing health against health. Indicators were considered for the model if: i) they fit into any one of the above socioeconomic or health priority areas; ii) showed significant statistical correlation with reductions in maternal and child mortality; iii) the statistical relationship was supported by the empirical and theoretical literature and; iv) at least 20% of data for the 21 years was available.

In fitting models of MMR, we were mindful of the extensive literature regarding the imprecision of MMR estimates, since maternal deaths are often underreported and both high and imprecisely measured in countries with the weakest health systems[[22](#_ENREF_22)]. Previous commentaries have alluded to the difficulty of fitting models to MMR estimates given this imprecision. For this reason, for the MMR model, only indicators with very strong association with maternal mortality were considered.

Based on the criteria described above we sequentially estimated over 100 different models and selected the model with the minimum Akaike Information Criterion. The Akaike information criteria (AIC) were noted and the covariate in each category that gave the best AIC was chosen. For some clusters, none of the variables yielded an improved AIC with significant coefficients. Therefore, stepwise backward elimination was then used to remove variables from the MMR or U5MR models that were not contributing to the predictive value of the model. For U5MR, this removed Log odds Skilled Birth Attendance (SBA). For MMR, it removed all variables except Log odds SBA, control of corruption index, Gini Coefficient, and square root TFR. For equivalent AICs the most parsimonious model was chosen. For example, although the governance indicator originally included in both models was the government effectiveness score, when the control of corruption index was included in its stead provided a better AIC and yielded more significant coefficients in the regression equation. It was thus kept and subsequent changes to the model was made with surviving variables in place. Lastly, where the distribution of the covariate of interest was skewed, transformations such as log odds and square root were tried as potential covariates for the same cluster. The final variables included in each model are shown by policy area in Table IIb. A sample of the types of iterative changes made for both the U5MR and MMR models are shown in Tables IIIa and IIIb respectively. The finals regressions used for the U5MR and MMR models are those highlighted in black color in column 13 in Table IIIa and column 11 in Table IIIb respectively.

Our final model included the following variables, all of which have statistical as well as theoretical and empirical support: percent of the population with access to clean water[[16](#_ENREF_16), [23-25](#_ENREF_23)], percent of children receiving the measles vaccine[[23](#_ENREF_23), [26-28](#_ENREF_26)], a control of corruption[[23](#_ENREF_23), [29](#_ENREF_29)], good governance[[20](#_ENREF_20)], power consumption per capita[[16](#_ENREF_16)], urbanization [[14](#_ENREF_14), [18](#_ENREF_18), [28-30](#_ENREF_28)], girls education [[18](#_ENREF_18), [19](#_ENREF_19), [31](#_ENREF_31), [32](#_ENREF_32)], total fertility rate[[23](#_ENREF_23), [24](#_ENREF_24), [33](#_ENREF_33), [34](#_ENREF_34)], physicians per capita [[17](#_ENREF_17), [18](#_ENREF_18), [23](#_ENREF_23), [26](#_ENREF_26), [31](#_ENREF_31), [35](#_ENREF_35), [36](#_ENREF_36)], percent of births attended by a skilled birth attendant[[23](#_ENREF_23), [26](#_ENREF_26), [37](#_ENREF_37)], health spending[[17-21](#_ENREF_17)], and gini coefficient[[26](#_ENREF_26), [37](#_ENREF_37), [38](#_ENREF_38)].

### Table IIa. Variables considered for inclusion in developing the fast-track target

| **Candidate Indicators** | | **Reason for In/Exclusion** |
| --- | --- | --- |
| 1 | Health Systems |  |
| 1a | Health Financing |  |
|  | Total health expenditure (government and non-government) | Not Stat. Sig. |
|  | Total health expenditure, (as % of GDP) | Endogenous |
|  | Total health expenditure per capita (current US$) | Endogenous |
|  | Total per capita health expenditure (PPP constant 2005 international $) | Endogenous |
|  | General Government health expenditure as % of total health expenditure | Endogenous |
|  | Government health expenditure as % of total government expenditure | Endogenous |
|  | ODA to maternal and newborn health per live birth | Limited data available for statistical analysis |
|  | ODA to child health per child under five | Limited data available for statistical analysis |
|  | Out-of-pocket-expenditure as (% of total health expenditure) | >80% of data available |
| 1b | Health Policies |  |
|  | National policy adopted on all provisions stipulated in the International Code of Marketing of Bre | Not appropriate for fixed effects model since it is a categorical variable and can only change once |
|  | National policy guidelines adopted on management of diarrhoea with low osmolarity oral rehydration | Not appropriate for fixed effects model since it is a categorical variable and can only change once |
|  | National policy adopted authorizing community health workers to identify and manage pneumonia with | Not appropriate for fixed effects model since it is a categorical variable and can only change once |
|  | National IMCI guidelines adapted to cover major conditions affecting newborn survival in the first | Not appropriate for fixed effects model since it is a categorical variable and can only change once |
|  | National plan or plans for scaling up maternal, newborn and child health interventions available a | Not appropriate for fixed effects model since it is a categorical variable and can only change once |
|  | National policy adopted authorizing midwives to administer obstetric and newborn health interventi | Not appropriate for fixed effects model since it is a categorical variable and can only change once |
|  | Legislation specifying grounds for which an abortion is permitted | Not appropriate for fixed effects model since it is a categorical variable and can only change once |
|  | Convention on the Rights of the Child (CRC) | Not appropriate for fixed effects model since it is a categorical variable and can only change once |
|  | Convention on the Elimination of all forms of Discrimination Against Women (CEDAW) | Not appropriate for fixed effects model since it is a categorical variable and can only change once |
|  | International Labour Organization Convention 183 ratified by the country or national legislatio | Not appropriate for fixed effects model since it is a categorical variable and can only change once |
| 1c | Health Information systems |  |
|  | Civil registration coverage (%): Births | Limited data available for statistical analysis |
|  | Civil registration coverage (%): Causes of death | Limited data available for statistical analysis |
| 1d | Human Resources (Health workforce) |  |
|  | Doctors (per 10,000 population) | >33% of data available |
|  | Nurses and midwives (per 10,000 population) | No data |
|  | Doctors/nurses/midwives (per 10,000 population) | No data |
|  | Nurses (per 1,000 population) | No data |
|  | Community health workers (per 10,000 population) | No data |
|  | Infrastructure: Hospital beds (per 10 000 population) | >33% of data available |
| 1e | Health Service coverage |  |
|  | Contraceptive prevalence (proportion of women aged 15-49 currently married who (or partner) are us | Limited data available for statistical analysis |
|  | Unmet need for family planning (proportion of women currently married that have an unmet need for | No data |
|  | Proportion of women attended at least once during pregnancy by skilled health personnel for reason | >33% of data available |
|  | Proportion of women attended at least four times during pregnancy by any provider (skilled or unsk | Limited data available for statistical analysis |
|  | Proportion of live births delivered by Caesarean section | Limited data available for statistical analysis |
|  | Proportion of live births attended by skilled health personnel | >33% of data available |
|  | Availability of emergency obstetric care services | No data |
|  | Proportion of newborns protected against tetanus | >80% of data available |
|  | Proportion of newborns put to the breast within one hour of birth | No data |
|  | Postnatal visit for baby (within 2 days for home births, %) | No data |
|  | Postnatal visit for mother (within 2 days for home births, %) | No data |
|  | Proportion of children ages 0–59 months with fever receiving any appropriate antimalarial drugs | Limited data available for statistical analysis |
|  | Proportion of children ages 0–59 months with suspected pneumonia taken to an appropriate health pr | Limited data available for statistical analysis |
|  | Proportion of children ages 0–59 months with suspected pneumonia receiving antibiotics | Limited data available for statistical analysis |
|  | Proportion of HIV-infected pregnant women who received antiretrovirals to reduce the risk of mothe | Limited data available for statistical analysis |
|  | Proportion of children ages 0–59 months sleeping under an insecticide-treated mosquito net | Limited data available for statistical analysis |
| 1f | Health technologies |  |
|  | Proportion of infants immunized with measles containing vaccine | >80% of data available |
|  | Proportion of infants who received three doses of diphtheria/ pertussis/ tetanus vaccine | >80% of data available |
|  | Proportion of infants who received three doses of Haemophilus influenzae type B vaccine | >80% of data available |
|  | Immunization, BCG (% of one-year-old children) | >80% of data available |
|  | Immunization, Pol3 (% of one-year-old children) | >80% of data available |
|  | MDG 8 Essential medicines: Median availability of selected generic medicines (Public), % | Limited data available for statistical analysis |
|  | MDG 8 Essential medicines: Median availability of selected generic medicines (Private), % | Limited data available for statistical analysis |
|  | MDG 8 Essential medicines: Median consumer price ratio of selected generic medicines (Public) | Limited data available for statistical analysis |
|  | MDG 8 Essential medicines: Median consumer price ratio of selected generic medicines (Private) | Limited data available for statistical analysis |
| **2** | **Education** |  |
|  | Expenditure per student, primary (% of GDP per capita) | >33% of data available |
|  | Primary education, pupils (% female) | No data |
|  | Adjusted net enrolment rate, primary, female (% of primary school age children) | >33% of data available |
|  | Expected years of schooling, male | >33% of data available |
|  | Expected years of schooling, female | >33% of data available |
|  | Adjusted net enrolment rate, primary (% of primary school age children) | >33% of data available |
|  | Female literacy rate (% aged 15-24) | No data |
|  | Adult literacy rate (%) | Limited data available for statistical analysis |
|  | Mean years of schooling (adults) (years) (aged 25 +) | Limited data available for statistical analysis |
|  | MDG 2 - Net primary school enrollment rate (%): Male | >33% of data available |
|  | MDG 2 - Net primary school enrollment rate (%): Female | >33% of data available |
|  | Global Innovation Index | Limited data available for statistical analysis |
|  | Public spending on education, total (% of GDP) | No data |
|  | Progression to secondary school (%) | No data |
|  | Number of scientific publications in the country | No data |
|  | Number of scientific publications in international collaboration | No data |
| 3 | Nutrition |  |
|  | Vitamin A supplementation coverage rate (% of children ages 6-59 months) | Can't regress health against health & Limited data available for statistical analysis |
|  | Proportion of children ages 0–59 months with diarrhoea receiving oral rehydration therapy and cont | Can't regress health against health & Limited data available for statistical analysis |
|  | Proportion of infants ages 0–5 months who are exclusively breastfed | Can't regress health against health & Limited data available for statistical analysis |
|  | Proportion of infants ages 6–8 months who are breastfed and introduced to solid food | Can't regress health against health & Limited data available for statistical analysis |
|  | Proportion of children ages 6–59 months who received two doses of vitamin A during the calendar ye | Can't regress health against health & Limited data available for statistical analysis |
|  | Proportion of children who are moderately or severely stunted | Can't regress health against health & Limited data available for statistical analysis |
|  | Proportion of children who are moderately or severely wasted | Can't regress health against health & Limited data available for statistical analysis |
|  | Proportion of children who are underweight for age | Can't regress health against health & Limited data available for statistical analysis |
|  | Proportion of low birthweight incidence (<2,500 g) | Can't regress health against health & Limited data available for statistical analysis |
|  | Prevalence of undernourishment (% of population) | Can't regress health against health |
|  | Malnutrition prevalence, weight for age (% of children under 5) | Can't regress health against health |
| 4 | Enviroment |  |
|  | Natural disasters (number of deaths) | No data |
|  | Natural disasters (population affected) | No data |
|  | Average precipitation in depth (mm per year) | No data |
|  | Proportion of the population using improved drinking water sources | >80% of data available |
|  | Improved sanitation facilities (% of population with access) | >80% of data available |
|  | Environmental Performance Index | Limited data available for statistical analysis |
| 5 | Infrastructure |  |
|  | Agricultural land (% of land area) | Not Stat. Sig. |
|  | Electric power consumption (kWh per capita) | >33% of data available |
|  | Mobile cellular subscriptions (per 100 people) | Limited data available for statistical analysis |
|  | Fixed broadband internet subscribers (per 100 people) | Limited data available for statistical analysis |
|  | Internet users (per 100 people) | Limited data available for statistical analysis |
|  | Telephone lines (per 100 people) | >80% of data available |
|  | Rail lines (total route-km) | Not Stat. Sig. |
|  | Road density (km of road per 100 sq. km of land area) | Collinear |
|  | Roads, paved (% of total roads) | >33% of data available |
|  | Roads, total network (km) | Not Stat. Sig. |
| 6 | Population Dynamics |  |
|  | Area (km2) | No variation in data - constant |
|  | Population: Total | Aweighing |
|  | Population density: Total population/area km2 | >80% of data available |
|  | Population: Median age (years) |  |
|  | Population: Aged under 15 (%) | >80 of data available |
|  | Population: Aged over 60 (%) | Endogns. |
|  | Population: Annual growth rate (%) |  |
|  | Population: Living in urban areas (%) |  |
|  | Adolescent fertility rate (per 1000 girls aged 15–19 years) |  |
|  | Birth rate, crude (per 1000 people) | Not relevent |
|  | Total fertility rate (children per women) | >80% of data available |
|  | Ethnic fractionalization | Limited data available for statistical analysis |
|  | Language fractionalization | No data |
|  | Religious fractionalization | No data |
| 7 | Inequality |  |
| 7a | Social Inequality |  |
|  | MDG 1 - Population living on <$1.25 (PPP int. $) a day (%) | >30% of data available |
|  | Equity - Concentration index for under-five mortality | Limited data available for statistical analysis |
|  | MDG 5 Births attended by skilled health personnel (%): Place of residence (Rural) | Limited data available for statistical analysis |
|  | MDG 5 Births attended by skilled health personnel (%): Place of residence (Urban) | Limited data available for statistical analysis |
|  | MDG 5 Births attended by skilled health personnel (%): Place of residence (Ratio urban–rural) | Limited data available for statistical analysis |
|  | MDG 5 Births attended by skilled health personnel (%): Place of residence (Difference urban–rural) | Limited data available for statistical analysis |
|  | MDG 5 Births attended by skilled health personnel (%): Wealth quintile (Lowest) | Limited data available for statistical analysis |
|  | MDG 5 Births attended by skilled health personnel (%): Wealth quintile (Highest) | Limited data available for statistical analysis |
|  | MDG 5 Births attended by skilled health personnel (%): Wealth quintile (Ratio highest–lowest) | Limited data available for statistical analysis |
|  | MDG 5 Births attended by skilled health personnel (%): Wealth quintile (Difference highest–lowest) | Limited data available for statistical analysis |
|  | MDG 5 Births attended by skilled health personnel (%): Educational level of mother (Lowest) | Limited data available for statistical analysis |
|  | MDG 5 Births attended by skilled health personnel (%): Educational level of mother (Highest) | Limited data available for statistical analysis |
|  | MDG 5 Births attended by skilled health personnel (%): Educational level of mother (Ratio highest– | Limited data available for statistical analysis |
|  | MDG 5 Births attended by skilled health personnel (%): Educational level of mother (Difference hig | Limited data available for statistical analysis |
|  | MDG 4 Measles immunization coverage among 1-year-olds (%): Place of residence (Rural) | Limited data available for statistical analysis |
|  | MDG 4 Measles immunization coverage among 1-year-olds (%): Place of residence (Urban) | Limited data available for statistical analysis |
|  | MDG 4 Measles immunization coverage among 1-year-olds (%): Place of residence (Ratio urban–rural) | Limited data available for statistical analysis |
|  | MDG 4 Measles immunization coverage among 1-year-olds (%): Place of residence (Difference urban–rural) | Limited data available for statistical analysis |
|  | MDG 4 Measles immunization coverage among 1-year-olds (%): Wealth quintile (Lowest) | Limited data available for statistical analysis |
|  | MDG 4 Measles immunization coverage among 1-year-olds (%): Wealth quintile (Highest) | Limited data available for statistical analysis |
|  | MDG 4 Measles immunization coverage among 1-year-olds (%): Wealth quintile (Ratio highest–lowest) | Limited data available for statistical analysis |
|  | MDG 4 Measles immunization coverage among 1-year-olds (%): Wealth quintile (Difference highest–lowest) | Limited data available for statistical analysis |
|  | National policy adopted authorizing community health workers to identify and manage pneumonia with antibiotics | Limited data available for statistical analysis |
|  | MDG 4 Measles immunization coverage among 1-year-olds (%): Educational level of mother (Highest) | Limited data available for statistical analysis |
|  | MDG 4 Measles immunization coverage among 1-year-olds (%): Educational level of mother (Ratio highest–lowest) | Limited data available for statistical analysis |
|  | MDG 4 Measles immunization coverage among 1-year-olds (%): Educational level of mother (Difference highest–lowest) | Limited data available for statistical analysis |
|  | MDG 4 Under-five mortality rate: Place of residence (Rural) | Limited data available for statistical analysis |
|  | MDG 4 Under-five mortality rate: Place of residence (Urban) | Limited data available for statistical analysis |
|  | MDG 4 Under-five mortality rate: Place of residence (Ratio urban–rural) | Limited data available for statistical analysis |
|  | MDG 4 Under-five mortality rate: Place of residence (Difference urban–rural) | Limited data available for statistical analysis |
|  | MDG 4 Under-five mortality rate: Wealth quintile (Lowest) | Limited data available for statistical analysis |
|  | MDG 4 Under-five mortality rate: Wealth quintile (Highest) | Limited data available for statistical analysis |
|  | MDG 4 Under-five mortality rate: Wealth quintile (Ratio highest–lowest) | Limited data available for statistical analysis |
|  | MDG 4 Under-five mortality rate: Wealth quintile (Difference highest–lowest) | Limited data available for statistical analysis |
|  | MDG 4 Under-five mortality rate: Educational level of mother (Lowest) | Limited data available for statistical analysis |
|  | MDG 4 Under-five mortality rate: Educational level of mother (Highest) | Limited data available for statistical analysis |
|  | MDG 4 Under-five mortality rate: Educational level of mother (Ratio highest–lowest) | Limited data available for statistical analysis |
|  | MDG 4 Under-five mortality rate: Educational level of mother (Difference highest–lowest) | Limited data available for statistical analysis |
|  | GINI Coefficient |  |
| 7b | Gender Inequality |  |
|  | Age at first marriage, female | Limited data available for statistical analysis |
|  | Women who were first married by 18 (% of women ages 20-24) | Limited data available for statistical analysis |
|  | Female legislators, senior officials and managers (% of total) | >33% of data available |
|  | Ratio of female to male labor participation rate (%) | Ratio value results in greater than 1 - conflict with model |
|  | Ratio of female to male primary enrollment (%) | Ratio value results in greater than 1 - conflict with model |
|  | Ratio of female to male secondary enrollment (%) | Ratio value results in greater than 1 - conflict with model |
|  | Ratio of female to male tertiary enrollment (%) | Ratio value results in greater than 1 - conflict with model |
|  | Ratio of young literate females to males (% age 15-24) | Ratio value results in greater than 1 - conflict with model |
|  | Labor force participation rate, female (% of female population ages 15-64) | >80% of data available |
|  | Share of women employed in the nonagricultural sector (% of total nonagricultural employment) | Collinear |
|  | Progression to secondary school, female (%) | >30% of data available |
|  | Labor force participation for ages 15-24, female (%) | >80% of data available |
|  | Gender Inequality Index | Limited data available for statistical analysis |
|  | Gender Empowerment Measure | No data |
|  | Gender Development Index | No variation in data: same all yrs |
| **8** | **Governance** |  |
|  | Women in the Upper House or Senate (%) | Limited data available for statistical analysis |
|  | Women in the lower or single House (%) | Limited data available for statistical analysis |
|  | Worldwide Governance Indicators (WGI): Voice and Accountability | >80 of data available |
|  | Worldwide Governance Indicators (WGI): Political Stability, No violence | >80 of data available |
|  | Worldwide Governance Indicators (WGI): Government effectiveness | >80 of data available |
|  | Worldwide Governance Indicators (WGI): Regulatory quality | >80 of data available |
|  | Worldwide Governance Indicators (WGI): Rule of Law | >80 of data available |
|  | Worldwide Governance Indicators (WGI): Control of corruption | >80 of data available |
|  | Democracy index | Limited data available for statistical analysis |
|  | Freedom of press index | gov_press |
|  | GLOBE Study (Regional index of leadership behaviors | Limited data available for statistical analysis |
|  | GLOBE Study (Regional index of leadership behaviors): Team oriented | Limited data available for statistical analysis |
|  | GLOBE Study (Regional index of leadership behaviors): Participative | Limited data available for statistical analysis |
|  | GLOBE Study (Regional index of leadership behaviors): Human oriented | Limited data available for statistical analysis |
|  | GLOBE Study (Regional index of leadership behaviors): Autonomous | Limited data available for statistical analysis |
|  | GLOBE Study (Regional index of leadership behaviors): Self-protection | Limited data available for statistical analysis |
|  | International Covenant on Economic, Social and Cultural Rights (CESCR) | No data |
| **9** | **Economic development** |  |
|  | Human Development Index | Collinear with GDP |
|  | Multidimensional Poverty Index | Collinear with GDP |
|  | Food imports (% of merchandise imports) | Collinear with GDP |
|  | GNI, Atlas method (current US$) | Collinear with GDP |
|  | Annual gross domestic product per capita | >80% of data available |
|  | Inflation, consumer prices (annual %) | Collinear with GDP |
|  | Labour force participation rate, total (% of total population ages 15-64) | Collinear with GDP |
|  | Coal rents (% of GDP) | Collinear with GDP |
|  | Mineral rents (% of GDP) | Collinear with GDP |
|  | Natural Gas rents (% of GDP) | Collinear with GDP |
|  | Net ODA received per capita (current US$) | Collinear with GDP |
|  | Net ODA received (% of GNI) | Collinear with GDP |
|  | Oil rents (% of GDP) | Collinear with GDP |
|  | Ores and metals exports (% of merchandise exports) | Collinear with GDP |
|  | Poverty headcount ratio at $1.25 a day (PPP) (% of population) | Collinear with GDP |
|  | Poverty headcount ratio at $2 a day (PPP) (% of population) | Collinear with GDP |
|  | Tariff rate, applied, simple mean, all products (%) | Collinear with GDP |
|  | Tax revenue (% of GDP) | Collinear with GDP |
|  | Unemployment, total (% of total labor force) | Collinear with GDP |
|  | Ease of Doing Business Index | Collinear with GDP |
|  | Employment in agriculture (% of total employment | Collinear with GDP |
|  | Arms imports (constant 1990 US$) | Collinear with GDP |
|  | Armed forces personnel (% of total labor force) | Collinear with GDP |
|  | Military expenditure (% of GDP) | Collinear with GDP |
|  | Labour force participation rate for ages 15-24, total (%) | Collinear with GDP |
| **10** | **Burden of disease** |  |
|  | Distribution of years of life lost by broader causes (%): Communicable | Can't regress health against health |
|  | Distribution of years of life lost by broader causes (%): Noncommunicable | Can't regress health against health |
|  | Distribution of years of life lost by broader causes (%): Injuries | Can't regress health against health |
|  | Communicable, maternal, perinatal and nutritional condition: GENERAL | Can't regress health against health |
|  | - Infectious and parasitic diseases | Can't regress health against health |
|  | - Respiratory infections | Can't regress health against health |
|  | - Maternal conditions | Can't regress health against health |
|  | - Perinatal conditions | Can't regress health against health |
|  | - Nutritional deficiencies | Can't regress health against health |
|  | Noncommunicable disease: GENERAL | Can't regress health against health |
|  | - Malignant neoplasms | Can't regress health against health |
|  | - Other neoplasms | Can't regress health against health |
|  | - Diabetes mellitus | Can't regress health against health |
|  | - Endocrine disorders | Can't regress health against health |
|  | - Neuropsychiatric conditions | Can't regress health against health |
|  | - Sense organ diseases | Can't regress health against health |
|  | - Cardiovascular diseases | Can't regress health against health |
|  | - Digestive diseases | Can't regress health against health |
|  | - Genitourinary diseases | Can't regress health against health |
|  | - Skin diseases | Can't regress health against health |
|  | - Musculoskeletal diseases | Can't regress health against health |
|  | - Congenital anomalies | Can't regress health against health |
|  | - Oral conditions | Can't regress health against health |
|  | Injuries: GENERAL | Can't regress health against health |
|  | - Injuries: Unintentional | Can't regress health against health |
|  | - Injuries: Intentional | Can't regress health against health |
|  | MDG 6 – AIDS, TB, Malaria |  |
|  | People living with HIV, 15-49 years old | Can't regress health against health |
|  | Notified cases of malaria per 100,000 population | Can't regress health against health |
|  | Tuberculosis prevalence rate per 100,000 population (mid-point) | Can't regress health against health |
|  | Incidence of tuberculosis (per 100,000 people) | Can't regress health against health |
|  | 4 and 5: Child and Maternal Mortality - IHME data | Can't regress health against health |
|  | Number of deaths of children less than five years of age per 1,000 live births | Can't regress health against health |
|  | Under-5 mortality average annual % change between 1990 and 2011 | Can't regress health against health |
|  | Measure of whether a country fulfils criteria for being "on track" to achieve MDG4 | Can't regress health against health |
|  | Number of maternal deaths per 100,000 live births | Can't regress health against health |
|  | Maternal mortality ratio average annual % change between 1990 and 2011 | Can't regress health against health |
|  | Measure of whether a country fulfils criteria for being "on track" to achieve MDG5a | Can't regress health against health |
|  | 4 and 5: Child and Maternal Mortality - UN Inter-agency data | Can't regress health against health |
|  | Number of deaths of children less than five years of age per 1,000 live births | Can't regress health against health |
|  | Under-5 mortality average annual % change between 1990 and 2010 | Can't regress health against health |
|  | Measure of whether a country fulfils criteria for being "on track" to achieve MDG4 (Countdown method) | Can't regress health against health |
|  | Stillbirth rate (per 1,000 total births) | Can't regress health against health |
|  | Infant mortality average annual % change between 1990 and 2010 | Can't regress health against health |
|  | Measure of whether a country fulfils criteria for being "on track" to achieve MDG4 | Can't regress health against health |
|  | Number of maternal deaths per 100,000 live births | Can't regress health against health |
|  | Maternal mortality ratio average annual % change between 1990 and 2010 | Can't regress health against health |
|  | Lifetime risk of maternal death (%) | Can't regress health against health |
|  | Measure of whether a country fulfils criteria for being "on track" to achieve MDG5a | Can't regress health against health |
|  | Contraceptive prevalence (proportion of women aged 15-49 currently married who (or partner) are using a contraceptive method (modern or traditional) | Can't regress health against health |
|  | Unmet need for family planning (proportion of women currently married) | Can't regress health against health |

### Table IIb. Variables included in U5MR and MMR models by priority area

## METHODS

### Overall Procedure

Both minimum and fast-track alternative targets were developed based on statistical models of U5MR and MMR to adjust for country-specific factors.

*Target 1 –the minimum performance target*– was based on a model of how each country’s time trend in reducing maternal and child mortality from 1990 to 2000 and projected GDP growth would affect the respective mortality rates in 2010.

*Target 2 –the fast-track target*— for U5MR and MMR was set based on a model of how a set of eight and four policy factors, respectively, in addition to past trends (time) and GDP affected the mortality rate. To meet Target 2, a country’s 2000-2010 actual health improvement had to exceed the health improvement that would have occurred had it accomplished the maximum improvement in each of the policy variables that was observed by fast-track performing countries in its sub-region between 2000 and 2010. The maximum improvement was different for each factor in each sub-region. Maximum improvement was defined by the largest proportional change in a factor or in the largest amount of the gap closed by all the countries in a sub-region over that decade, and this proportional change was applied to the level of each factor in 2000 to calculate each country’s fast-track target for 2010.

### Statistical Model

A fixed effects model was selected for analysis of U5MR on theoretical and statistical grounds. Fixed effects models are more appropriate for analyses of panel data on countries than are random effects models.[[39](#_ENREF_39)] Over time countries have durable socio-demographic features including political instability that are confounded with policy variables. Fixed effects models with longitudinal data control for unmeasured time-invariant confounders which may cause a country to do well or poorly (such as language fractionalization, culture, or geographic region), and permit each country to have its own intercept. However, to test the appropriateness of this model we ran our models with both random and fixed effects, and performed a Hausman test, which was significant (P < .001) thus favoring the fixed effects model.^3^ Regression equations are shown in the appendix Table IV.

The fixed effects models were run using the natural logarithm of U5MR as the dependent variable. For both U5MR and MMR, the untransformed dependent variables had a skewed distribution, while the log-transformed data were more symmetric and approximately normal. Additionally, many of the independent variables in our analysis also had a skewed distribution, and showed a clear but non-linear relationship to the outcome variable of interest. In all these cases, the independent variables were transformed to make their distributions more symmetric and linearly related to the outcome. Transformations used for this purpose were logarithm, square root, and log odds [log odds = log $(\frac{p}{1-p})$ ]. For log odds, since many values in our dataset were at a cutoff value (e.g. 0 or 100%), all such data to be transformed by log odds were shifted away from the cutoff by 0.5% in order to permit a log odds transformation. Imputations were performed using the transformed data in all cases. Fixed effects regressions for log(U5MR) were then performed using the log-linear transform equations (see regressions section below for equation and appendix Tables IVb and IVb for regressions output).

For MMR, data were much more sparse; four countries lacked any national reported estimates, and 5 additional countries lacked UNFPA modeled estimates, [[40](#_ENREF_40)] and were thus excluded from the analysis. Another 21 countries had only one data point reported, rendering these countries inappropriate for a fixed effects model. In order to include as many countries as possible, ordinary least squares regression (OLS) adjusting standard errors for clustering was employed for MMR.

### Regression Equation

The general regression equations for the fixed effects (FE) model for U5MR is Log(U5MR)_it_ = X_it_β + α_I_ + ε_it_. The general regression equations for the Ordinary Least Squares (OLS) models for MMR is log(MMR) _it_= X_it_β + ε_it_.

The FE model used Log(U5MR) as the independent variable and Log(GDP per capita), time, and 8 policy variables as the independent variables. These 8 policy variables (all included in Table IVa below), were: Log odds of having access to improved water source, Log odds of a child under 2 having the Measles vaccine, control of corruption index, percent urbanized, Log of Health Spending per capita lagged by 5 years, Log Power Consumption (KWh per capita), square root Doctors per 100,000 population, and Log odds of Girls Enrolled in Primary School. The equation reads as shown. Terms in bold are vectors or matrices, i indicates each individual country (of 144 total), and t indicates the 22 years in our dataset (from 1990-2011, t = 0 indicates 1990):

Log(U5MR)_it_ = β_0_ + β_1_*Log(GDP per capita)_it_ + β_2_*time + **X_it_·β** + αi + εit (1)

All of the above terms on the right side of the equation are known except for α_i_. α_i_ represents the addition of other, unmeasured factors which may shift a country’s U5MR, such as region, culture, or language fractionalization. Assuming these factors are time invariant, which is certainly reasonable for region and probably for culture over such a short time period, it is possible to eliminate them by taking a fixed effects model, subtracting the mean from both sides of the equation. Fixed effects models calculate the mean of each term for each individual country and subtracts it from each data point. For time varying terms, which includes all except for α_i_, this does not change the magnitude of the coefficient. However, since the mean of all α_i_ for a given country is α_i_, the effect of this term disappears. The theoretical version of the fixed effects model, showing how the fixed effects are eliminated by taking the difference of the original equation from its mean is shown here:

$y$_it_ - $\bar{y}$ = (**X­_it_** - $\bar{\mathbf{X}}$**)·β** + (α_i_ - $\bar{}$_i_) + (ε_it_ - $\bar{}$) = (**X­_it_** - $\bar{\mathbf{X}}$**)·β** + ε_it_ (2)

A fixed effects model for MMR was not possible since there were 21 countries in our dataset with only 1 reported value. Thus, Ordinary Least Squares (OLS) regression adjusting standard errors for clustering was used, see Table IVb. In OLS regression, all errors are assumed to be independent. Such an assumption is not appropriate for values reported from individual countries; they are likely to be correlated with each other. Robust clustering adjusts the standard errors of the coefficients in the OLS equation to account for this non-independence. Besides this adjustment, our MMR model was a simple OLS model, controlling for GDP, time, and 4 policy variables: control of corruption index, Log Odds of Skilled Birth Attendance, square root of Total Fertility Rate, and Gini coefficient. The i subscript indicates each individual country (of 116 total) and t indicates a time variable covering the 22 years in our dataset (from 1990-2011, t = 0 indicates 1990). The equation is indicated thusly:

Log(MMR)_it_ = β_0_ + β_1_*Log(GDP per capita)_t_ + β_2_*time + **X_*_β** + ε_it_ (3)

The MMR targets for each individual country’s curve were projected with the reference point being the UNFPA modeled estimates for the earliest year that data was available for that country, which was 1990 for 104 countries, 1995 for 9 others, and 2000 for 3 countries.

**How Regression Lowers Uncertainty around a Noisy Estimate**

Suppose in country i at time t, the true mortality is U5MR**_it_*. The UNICEF demographers will have obtained a noisy estimate for this value from a household survey like MICS or DHS that we will call U5MR*_it_*. The measurement error can be modeled as the difference between the truth and the noisy estimate: U5MR***­_it_-** U5MR_it_ *=ε_it_*. We assume that the noise is random.

To produce a modeled estimate the demographers first estimate an equation using the noisy data such as: *U5MR_it_=C+β_2_TimeSpline+μ_i_+ε_it_* (4)

Where μ_i_ is a fixed effect for each country. Then they use the regression model to calculate:

*Modeled_U5MR_it_=C+β_1_X_it_+β_2_TimeSpline*  (5)

It has long been known that the uncertainty around *Modeled_U5MR_it_* will be much less than the uncertainty around *U5MR_it_*  because Equation [2] does not have the error terms in it. [[41](#_ENREF_41)]

All calculations were performed in Stata version 12.[[42](#_ENREF_42)] All GDP figures are in constant 2000 USD.

###

### Building the Targets

To construct Target 1, the “minimum performance” target, each country’s predicted value of log(U5MR) or log(MMR) was produced for 2010 based on the model and the projected 2010 value of GDP, given each country’s starting point in 2000. GDP was projected to 2010 based on the ten year average GDP growth rate from 1990 to 2000. Projected GDP was used to stay in keeping with the principle that these would be targets that would need to be set without certainty about the economic future. Targets set using the projected and actual GDP in 2010 were very similar (data not shown). The predicted value of 2010 log(U5MR) or log(MMR) was exponentiated to obtain a prediction in the original units, and then adjusted using Duan’s smearing estimator.[[43](#_ENREF_43)]

In order to determine Target 2, the “fast-track” target, the best performance for each of the independent variables except GDP was determined by sub-region. For variables whose metric was a quantity per capita (doctors, power consumption, and lag of health spending) there is no logical upper or lower limit, so best performance was considered to be the highest percent increase from 2000 to 2010 relative to the all other countries in the related geographical sub-region. For all other variables, the ideal performance occurs at some upper or lower limit (e.g. the best performance of the access to clean water variable is 100%), so the best performance from 2000 to 2010 was considered to be the percent of the gap closed for that specific indicator from 2000 to 2010. Basing ideal performance on gap closure was more appropriate than basing the ideal on an attained level since it would not impose that countries doing very poorly in 2000 be held to an impossible standard if another country in their region was already doing particularly well. For example, a country that increases its percent of people with access to clean water from 62% to 83% from 2000-2010, as Malawi did, is considered the best performer in East Africa, despite the fact that Mauritius, in the same sub-region, already had 99% access in 2000.

Once these best performances were calculated by sub-region, the fast-track targets were created analogously to the minimum performance targets. Log(U5MR) and Log(MMR) were predicted for 2010 using the projected GDP as well as the hypothetical scenario that each country performed as well as the observed regional best performance on each factor in the respective U5MR and MMR models from 2000-2010. In other words, countries were given a stringent hurdle of needing to maximize performance on multiple relevant factors, being compared against the best performer on each factor in their region.

### Table IIIa. Variables and regressions test for the U5MR model

### Table IIIb. Variables and regressions test for the MMR model

# SECTION 2 - RESULTS

### Table IVa. Regression results - U5MR targets

### Table IVb. Regression results - MMR targets

# SECTION 3 - UNCERTAINTY ANALYSIS

### Imputation

The model used to establish both low and fast-track targets was based on data that were available for 144 and 116 countries for MDG 4 and 5 respectively and historical data for some key indicators did not go back before 1995. We used standard imputation methods to address this data limitation and in sensitivity tests we show how the use of imputation would not have a large effect on the position of the fast-track target.

### Weighting to Make a Global Aggregate

According to epidemiological logic weighting each country’s U5MR and MMR by the size of each country’s birth cohort is the appropriate way to make a global aggregate to track MDG 4 and 5 for the globe. One would hold that since these are epidemiological risks per birth or pregnancy they should be weighted by the number of those exposed to childbirth and/or pregnancy. However population weighting could also be advocated on the grounds that these indicators are part of the social welfare of all members of a country’s population due to widely shared concern for the well-being of women and children and the significant social and economic benefits resulting from their lives saved. A country with a large population would have many more people to experience concern about the levels of U5MR and MMR even if it had only a few births. For these reasons we present both approaches and prefer population weighting.

### Uncertainty Range

To generate an uncertainty range, each target was considered to be the sum of a linear combination of stochastic contributions i.e. Target=C+β_1_X_1_+β_2_X_2_+β_3_X_3_+…+ε where each β was modeled as a normally distributed variable with mean and standard deviation as estimated in the original regression model. We performed a Monte Carlo estimate in which the targets were recalculated 100 times and these iterations produced an estimated mean and standard deviation. The uncertainty ranges are shown in the appendix Table V, and are narrow and symmetric enough to provide confidence that the calculated targets are appropriate for the analyses described given the data limitations.

### Table V. Uncertainty analysis ranges

|  |  |  |  |  |  |  |  |  |  |  |  |  |  |  |  |  |  |  |  |  |  |  |
| --- | --- | --- | --- | --- | --- | --- | --- | --- | --- | --- | --- | --- | --- | --- | --- | --- | --- | --- | --- | --- | --- | --- |
|  | **Country** |  | **Under-5 Mortality** | | | | | | | | |  | **Maternal Mortality** | | | | | | | | |  |
|  |  |  | Minimum Performance Target | | | |  | Fast-Track Target | | | |  | Minimum Performance Target | | | |  | Fast-Track Target | | | |  |
|  |  |  | mean | 95% C.I. Range | | |  | mean | 95% C.I. Range | | |  | mean | Range | | |  | mean | 95% C.I. Range | | |  |
|  | Albania |  | 17.9 | 14.7 | to | 21.1 |  | 15.4 | 10.2 | to | 20.6 |  | - | - |  | - |  | - | - |  | - |  |
|  | Algeria |  | 33.5 | 26.3 | to | 40.7 |  | 28.9 | 18.7 | to | 39.1 |  | 161.1 | 135.2 | to | 186.9 |  | 75.1 | 55.0 | to | 95.3 |  |
|  | Angola |  | 147.6 | 124.0 | to | 171.2 |  | 119.5 | 83.5 | to | 155.5 |  | - | - |  | - |  | - | - |  | - |  |
|  | Argentina |  | 13.9 | 10.5 | to | 17.3 |  | 12.3 | 5.5 | to | 19.1 |  | 36.0 | 30.4 | to | 41.7 |  | 27.0 | 18.5 | to | 35.5 |  |
|  | Armenia |  | 21.7 | 18.1 | to | 25.3 |  | 17.5 | 9.9 | to | 25.1 |  | 33.3 | 27.4 | to | 39.2 |  | 22.7 | 14.5 | to | 31.0 |  |
|  | Azerbaijan |  | 51.4 | 43.0 | to | 59.8 |  | 39.9 | 23.3 | to | 56.5 |  | 50.4 | 42.0 | to | 58.8 |  | 28.7 | 15.7 | to | 41.6 |  |
|  | Bangladesh |  | 58.3 | 49.1 | to | 67.5 |  | 47.0 | 33.2 | to | 60.8 |  | 391.8 | 316.1 | to | 467.4 |  | 221.8 | 116.3 | to | 327.4 |  |
|  | Belarus |  | 8.5 | 6.7 | to | 10.3 |  | 7.8 | 4.4 | to | 11.2 |  | - | - |  | - |  | - | - |  | - |  |
|  | Belize |  | 18.9 | 15.1 | to | 22.7 |  | 16.4 | 9.8 | to | 23.0 |  | 38.9 | 32.2 | to | 45.5 |  | 18.3 | 13.4 | to | 23.1 |  |
|  | Benin |  | 101.5 | 84.3 | to | 118.7 |  | 84.7 | 61.1 | to | 108.3 |  | 496.4 | 424.5 | to | 568.2 |  | 169.5 | 81.1 | to | 257.8 |  |
|  | Bhutan |  | 58.7 | 47.1 | to | 70.3 |  | 49.7 | 35.5 | to | 63.9 |  | 367.6 | 263.2 | to | 471.9 |  | 137.9 | 83.9 | to | 191.9 |  |
|  | Bolivia |  | 58.2 | 45.8 | to | 70.6 |  | 43.1 | 28.9 | to | 57.3 |  | 274.3 | 232.5 | to | 316.1 |  | 112.9 | 61.6 | to | 164.2 |  |
|  | Bosnia and Herzegovina |  | 6.0 | 4.6 | to | 7.4 |  | 5.6 | 3.4 | to | 7.8 |  | - | - |  | - |  | - | - |  | - |  |
|  | Botswana |  | 38.5 | 30.1 | to | 46.9 |  | 35.6 | 22.0 | to | 49.2 |  | 69.5 | 57.2 | to | 81.7 |  | 40.7 | 29.0 | to | 52.5 |  |
|  | Brazil |  | 24.5 | 19.3 | to | 29.7 |  | 20.1 | 11.9 | to | 28.3 |  | 77.7 | 63.6 | to | 91.8 |  | 40.5 | 29.0 | to | 52.0 |  |
|  | Bulgaria |  | 14.1 | 11.3 | to | 16.9 |  | 12.7 | 7.1 | to | 18.3 |  | - | - |  | - |  | - | - |  | - |  |
|  | Burkina Faso |  | 127.9 | 107.1 | to | 148.7 |  | 106.4 | 81.2 | to | 131.6 |  | 389.0 | 319.0 | to | 459.0 |  | 128.0 | 67.4 | to | 188.6 |  |
|  | Burundi |  | 129.0 | 110.8 | to | 147.2 |  | 100.1 | 76.1 | to | 124.1 |  | 1166.0 | 967.6 | to | 1364.4 |  | 300.2 | 165.0 | to | 435.3 |  |
|  | Cambodia |  | 59.2 | 49.6 | to | 68.8 |  | 41.7 | 31.1 | to | 52.3 |  | 307.5 | 239.9 | to | 375.1 |  | 219.9 | 136.9 | to | 302.9 |  |
|  | Cameroon |  | 106.7 | 90.7 | to | 122.7 |  | 82.4 | 57.8 | to | 107.0 |  | 572.3 | 487.2 | to | 657.5 |  | 298.1 | 183.2 | to | 412.9 |  |
|  | Cape Verde |  | 25.6 | 20.4 | to | 30.8 |  | 21.7 | 14.1 | to | 29.3 |  | 84.3 | 70.4 | to | 98.2 |  | 38.5 | 22.4 | to | 54.7 |  |
|  | Central African Republic |  | 131.2 | 112.0 | to | 150.4 |  | 98.8 | 75.2 | to | 122.4 |  | 844.0 | 709.3 | to | 978.6 |  | 418.2 | 248.9 | to | 587.5 |  |
|  | Chad |  | 144.5 | 122.7 | to | 166.3 |  | 109.0 | 78.2 | to | 139.8 |  | 646.9 | 443.8 | to | 850.1 |  | 312.6 | 155.5 | to | 469.6 |  |
|  | Chile |  | 8.1 | 6.1 | to | 10.1 |  | 6.6 | 3.8 | to | 9.4 |  | 23.7 | 19.8 | to | 27.6 |  | 14.7 | 10.9 | to | 18.5 |  |
|  | China |  | 19.6 | 15.4 | to | 23.8 |  | 18.6 | 11.8 | to | 25.4 |  | 27.2 | 20.7 | to | 33.8 |  | 52.8 | 36.7 | to | 68.9 |  |
|  | Colombia |  | 18.3 | 14.5 | to | 22.1 |  | 13.9 | 7.9 | to | 19.9 |  | 108.2 | 88.1 | to | 128.2 |  | 76.9 | 43.6 | to | 110.2 |  |
|  | Comoros |  | 76.2 | 64.2 | to | 88.2 |  | 57.7 | 41.3 | to | 74.1 |  | 397.8 | 328.7 | to | 466.9 |  | 165.5 | 87.0 | to | 244.1 |  |
|  | Congo, Dem. Rep. |  | 153.7 | 136.1 | to | 171.3 |  | 117.5 | 91.3 | to | 143.7 |  | 1686.3 | 1384.6 | to | 1988.0 |  | 541.2 | 251.8 | to | 830.6 |  |
|  | Congo, Rep. |  | 83.3 | 67.1 | to | 99.5 |  | 65.9 | 45.3 | to | 86.5 |  | 346.7 | 291.1 | to | 402.3 |  | 239.0 | 161.3 | to | 316.7 |  |
|  | Costa Rica |  | 9.3 | 7.3 | to | 11.3 |  | 7.8 | 4.2 | to | 11.4 |  | 20.3 | 16.4 | to | 24.3 |  | 11.8 | 8.5 | to | 15.1 |  |
|  | Cote d'Ivoire |  | 104.7 | 87.3 | to | 122.1 |  | 82.9 | 56.7 | to | 109.1 |  | 599.9 | 482.7 | to | 717.1 |  | 210.4 | 104.7 | to | 316.0 |  |
|  | Croatia |  | 5.8 | 4.6 | to | 7.0 |  | 5.0 | 2.8 | to | 7.2 |  | - | - |  | - |  | - | - |  | - |  |
|  | Cuba |  | 6.5 | 4.9 | to | 8.1 |  | 5.4 | 2.6 | to | 8.2 |  | 40.3 | 34.1 | to | 46.6 |  | 28.4 | 21.0 | to | 35.8 |  |
|  | Czech Republic |  | 4.8 | 3.6 | to | 6.0 |  | 4.3 | 2.3 | to | 6.3 |  | - | - |  | - |  | - | - |  | - |  |
|  | Djibouti |  | 83.9 | 69.1 | to | 98.7 |  | 60.6 | 36.8 | to | 84.4 |  | 294.7 | 241.1 | to | 348.2 |  | 101.0 | 55.7 | to | 146.4 |  |
|  | Dominica |  | 10.7 | 8.1 | to | 13.3 |  | 9.5 | 5.1 | to | 13.9 |  | - | - |  | - |  | - | - |  | - |  |
|  | Dominican Republic |  | 26.9 | 21.5 | to | 32.3 |  | 22.0 | 13.6 | to | 30.4 |  | 99.0 | 77.2 | to | 120.7 |  | 68.7 | 39.6 | to | 97.9 |  |
|  | Ecuador |  | 25.0 | 20.2 | to | 29.8 |  | 19.4 | 11.4 | to | 27.4 |  | 123.3 | 103.9 | to | 142.7 |  | 60.6 | 29.3 | to | 91.9 |  |
|  | Egypt, Arab Rep. |  | 31.1 | 25.1 | to | 37.1 |  | 25.1 | 15.7 | to | 34.5 |  | 124.2 | 102.3 | to | 146.2 |  | 89.5 | 68.9 | to | 110.2 |  |
|  | El Salvador |  | 22.4 | 17.2 | to | 27.6 |  | 19.1 | 10.9 | to | 27.3 |  | 77.2 | 63.1 | to | 91.3 |  | 50.3 | 31.1 | to | 69.4 |  |
|  | Equatorial Guinea |  | 85.6 | 66.6 | to | 104.6 |  | 75.4 | 44.0 | to | 106.8 |  | - | - |  | - |  | - | - |  | - |  |
|  | Eritrea |  | 72.1 | 62.3 | to | 81.9 |  | 57.1 | 43.7 | to | 70.5 |  | 332.5 | 286.7 | to | 378.3 |  | 100.7 | 58.8 | to | 142.7 |  |
|  | Estonia |  | 6.2 | 4.6 | to | 7.8 |  | 5.8 | 3.6 | to | 8.0 |  | - | - |  | - |  | - | - |  | - |  |
|  | Ethiopia |  | 96.1 | 81.9 | to | 110.3 |  | 74.5 | 60.1 | to | 88.9 |  | 603.0 | 512.1 | to | 694.0 |  | 178.6 | 93.4 | to | 263.8 |  |
|  | Fiji |  | 16.4 | 12.8 | to | 20.0 |  | 15.6 | 10.0 | to | 21.2 |  | 21.0 | 17.7 | to | 24.4 |  | 13.6 | 10.9 | to | 16.3 |  |
|  | Gabon |  | 61.8 | 47.4 | to | 76.2 |  | 48.5 | 28.5 | to | 68.5 |  | 228.8 | 192.6 | to | 265.0 |  | 164.9 | 112.4 | to | 217.4 |  |
|  | Gambia, The |  | 96.1 | 80.1 | to | 112.1 |  | 80.5 | 52.1 | to | 108.9 |  | 519.3 | 422.5 | to | 616.2 |  | 212.9 | 117.1 | to | 308.7 |  |
|  | Georgia |  | 24.9 | 20.7 | to | 29.1 |  | 19.8 | 13.0 | to | 26.6 |  | 95.2 | 80.0 | to | 110.4 |  | 38.7 | 24.9 | to | 52.5 |  |
|  | Ghana |  | 70.8 | 60.4 | to | 81.2 |  | 55.6 | 39.2 | to | 72.0 |  | 330.1 | 267.0 | to | 393.3 |  | 114.3 | 65.9 | to | 162.8 |  |
|  | Grenada |  | 11.4 | 8.6 | to | 14.2 |  | 10.1 | 6.1 | to | 14.1 |  | 18.7 | 15.9 | to | 21.5 |  | 8.7 | 6.2 | to | 11.2 |  |
|  | Guatemala |  | 35.1 | 28.9 | to | 41.3 |  | 29.3 | 18.5 | to | 40.1 |  | 100.0 | 80.8 | to | 119.3 |  | 51.7 | 32.3 | to | 71.0 |  |
|  | Guinea |  | 128.7 | 106.3 | to | 151.1 |  | 102.5 | 75.7 | to | 129.3 |  | 893.9 | 746.6 | to | 1041.2 |  | 315.8 | 167.6 | to | 464.0 |  |
|  | Guinea-Bissau |  | 144.2 | 123.6 | to | 164.8 |  | 110.1 | 80.1 | to | 140.1 |  | 928.5 | 772.3 | to | 1084.8 |  | 282.3 | 144.1 | to | 420.5 |  |
|  | Guyana |  | 33.7 | 27.7 | to | 39.7 |  | 26.3 | 17.7 | to | 34.9 |  | 79.2 | 63.3 | to | 95.0 |  | 48.5 | 27.1 | to | 69.8 |  |
|  | Haiti |  | 79.8 | 67.8 | to | 91.8 |  | 60.8 | 46.2 | to | 75.4 |  | 462.1 | 359.3 | to | 564.9 |  | 65.1 | 11.7 | to | 118.4 |  |
|  | Honduras |  | 25.3 | 21.1 | to | 29.5 |  | 21.8 | 14.4 | to | 29.2 |  | 147.5 | 123.8 | to | 171.1 |  | 66.8 | 40.6 | to | 93.1 |  |
|  | Hungary |  | 7.5 | 5.9 | to | 9.1 |  | 6.8 | 3.6 | to | 10.0 |  | - | - |  | - |  | - | - |  | - |  |
|  | India |  | 59.5 | 48.7 | to | 70.3 |  | 48.3 | 33.5 | to | 63.1 |  | 260.8 | 211.7 | to | 310.0 |  | 141.5 | 85.0 | to | 197.9 |  |
|  | Indonesia |  | 36.6 | 29.2 | to | 44.0 |  | 26.5 | 16.7 | to | 36.3 |  | 295.5 | 248.3 | to | 342.6 |  | 284.4 | 194.4 | to | 374.3 |  |
|  | Iran, Islamic Rep. |  | 29.7 | 23.9 | to | 35.5 |  | 24.4 | 14.0 | to | 34.8 |  | 35.6 | 25.9 | to | 45.4 |  | 18.8 | 10.2 | to | 27.4 |  |
|  | Iraq |  | 30.8 | 24.8 | to | 36.8 |  | 22.3 | 12.7 | to | 31.9 |  | 61.2 | 52.0 | to | 70.4 |  | 17.7 | 7.3 | to | 28.1 |  |
|  | Jamaica |  | 18.6 | 14.8 | to | 22.4 |  | 15.3 | 9.5 | to | 21.1 |  | 38.9 | 33.1 | to | 44.8 |  | 28.4 | 16.9 | to | 39.9 |  |
|  | Jordan |  | 20.6 | 16.4 | to | 24.8 |  | 16.8 | 9.6 | to | 24.0 |  | 69.5 | 57.0 | to | 82.0 |  | 18.8 | 11.4 | to | 26.2 |  |
|  | Kazakhstan |  | 31.2 | 25.4 | to | 37.0 |  | 27.5 | 16.1 | to | 38.9 |  | 54.3 | 44.8 | to | 63.8 |  | 52.5 | 41.7 | to | 63.2 |  |
|  | Kenya |  | 76.7 | 62.9 | to | 90.5 |  | 62.0 | 44.2 | to | 79.8 |  | 329.8 | 261.0 | to | 398.6 |  | 121.3 | 67.8 | to | 174.8 |  |
|  | Kiribati |  | 47.9 | 37.9 | to | 57.9 |  | 42.9 | 27.1 | to | 58.7 |  | - | - |  | - |  | - | - |  | - |  |
|  | Kyrgyz Republic |  | 35.5 | 29.9 | to | 41.1 |  | 29.7 | 20.3 | to | 39.1 |  | 73.0 | 61.6 | to | 84.4 |  | 45.9 | 34.5 | to | 57.4 |  |
|  | Lao PDR |  | 54.3 | 45.7 | to | 62.9 |  | 36.4 | 24.4 | to | 48.4 |  | 694.0 | 538.3 | to | 849.6 |  | 312.7 | 194.0 | to | 431.4 |  |
|  | Latvia |  | 11.5 | 8.7 | to | 14.3 |  | 11.8 | 7.4 | to | 16.2 |  | - | - |  | - |  | - | - |  | - |  |
|  | Lebanon |  | 12.6 | 9.6 | to | 15.6 |  | 10.4 | 5.2 | to | 15.6 |  | 21.0 | 16.1 | to | 25.8 |  | 18.2 | 12.8 | to | 23.6 |  |
|  | Lesotho |  | 74.9 | 61.3 | to | 88.5 |  | 66.1 | 46.9 | to | 85.3 |  | 299.3 | 166.1 | to | 432.5 |  | 171.5 | 106.7 | to | 236.3 |  |
|  | Liberia |  | 105.4 | 90.0 | to | 120.8 |  | 84.7 | 61.9 | to | 107.5 |  | 802.0 | 677.6 | to | 926.5 |  | 283.4 | 157.4 | to | 409.5 |  |
|  | Libya |  | 18.7 | 14.1 | to | 23.3 |  | 16.6 | 9.4 | to | 23.8 |  | 52.6 | 45.0 | to | 60.2 |  | 43.3 | 33.2 | to | 53.3 |  |
|  | Lithuania |  | 8.5 | 6.7 | to | 10.3 |  | 8.1 | 4.9 | to | 11.3 |  | - | - |  | - |  | - | - |  | - |  |
|  | Macedonia, FYR |  | 13.4 | 10.8 | to | 16.0 |  | 11.4 | 6.4 | to | 16.4 |  | - | - |  | - |  | - | - |  | - |  |
|  | Madagascar |  | 76.9 | 65.1 | to | 88.7 |  | 59.1 | 42.9 | to | 75.3 |  | 568.8 | 477.2 | to | 660.3 |  | 158.6 | 90.8 | to | 226.4 |  |
|  | Malawi |  | 110.7 | 95.9 | to | 125.5 |  | 90.8 | 69.0 | to | 112.6 |  | 674.4 | 553.7 | to | 795.2 |  | 180.2 | 96.4 | to | 263.9 |  |
|  | Malaysia |  | 7.2 | 5.6 | to | 8.8 |  | 5.1 | 2.7 | to | 7.5 |  | 23.5 | 18.7 | to | 28.2 |  | 19.9 | 13.5 | to | 26.2 |  |
|  | Maldives |  | 24.8 | 18.8 | to | 30.8 |  | 20.4 | 12.8 | to | 28.0 |  | 118.7 | 95.9 | to | 141.6 |  | 74.0 | 46.8 | to | 101.3 |  |
|  | Mali |  | 157.1 | 135.3 | to | 178.9 |  | 129.0 | 96.0 | to | 162.0 |  | 687.7 | 564.3 | to | 811.2 |  | 243.7 | 134.1 | to | 353.2 |  |
|  | Marshall Islands |  | 27.7 | 22.1 | to | 33.3 |  | 26.1 | 15.1 | to | 37.1 |  | - | - |  | - |  | - | - |  | - |  |
|  | Mauritania |  | 88.7 | 73.9 | to | 103.5 |  | 71.3 | 48.3 | to | 94.3 |  | 536.8 | 425.2 | to | 648.4 |  | 199.5 | 111.7 | to | 287.4 |  |
|  | Mauritius |  | 12.6 | 9.8 | to | 15.4 |  | 10.8 | 5.8 | to | 15.8 |  | 30.7 | 24.7 | to | 36.6 |  | 23.6 | 17.9 | to | 29.3 |  |
|  | Mexico |  | 20.7 | 15.3 | to | 26.1 |  | 18.3 | 9.5 | to | 27.1 |  | 57.3 | 46.8 | to | 67.8 |  | 36.2 | 26.6 | to | 45.8 |  |
|  | Micronesia, Fed. Sts. |  | 36.4 | 29.0 | to | 43.8 |  | 31.2 | 19.0 | to | 43.4 |  | 93.2 | 77.4 | to | 109.0 |  | 91.2 | 70.6 | to | 111.7 |  |
|  | Moldova |  | 19.3 | 16.7 | to | 21.9 |  | 15.4 | 10.4 | to | 20.4 |  | - | - |  | - |  | - | - |  | - |  |
|  | Mongolia |  | 43.9 | 36.5 | to | 51.3 |  | 40.7 | 24.1 | to | 57.3 |  | 85.2 | 69.7 | to | 100.7 |  | 45.6 | 33.8 | to | 57.5 |  |
|  | Montenegro |  | 8.6 | 6.6 | to | 10.6 |  | 7.5 | 4.5 | to | 10.5 |  | - | - |  | - |  | - | - |  | - |  |
|  | Morocco |  | 37.8 | 30.0 | to | 45.6 |  | 31.6 | 19.8 | to | 43.4 |  | 184.8 | 154.4 | to | 215.2 |  | 99.3 | 75.6 | to | 123.0 |  |
|  | Mozambique |  | 114.6 | 95.6 | to | 133.6 |  | 96.0 | 68.6 | to | 123.4 |  | 463.7 | 387.7 | to | 539.8 |  | 185.5 | 112.9 | to | 258.2 |  |
|  | Namibia |  | 48.5 | 38.1 | to | 58.9 |  | 43.2 | 26.8 | to | 59.6 |  | 128.0 | 85.2 | to | 170.9 |  | 74.1 | 52.4 | to | 95.8 |  |
|  | Nepal |  | 58.4 | 49.2 | to | 67.6 |  | 48.1 | 36.7 | to | 59.5 |  | 434.7 | 363.4 | to | 506.0 |  | 166.8 | 95.5 | to | 238.2 |  |
|  | Nicaragua |  | 30.2 | 24.8 | to | 35.6 |  | 26.1 | 17.5 | to | 34.7 |  | 110.1 | 90.6 | to | 129.5 |  | 52.3 | 31.5 | to | 73.2 |  |
|  | Niger |  | 160.9 | 133.7 | to | 188.1 |  | 131.3 | 105.1 | to | 157.5 |  | 1069.8 | 875.8 | to | 1263.8 |  | 302.8 | 127.2 | to | 478.3 |  |
|  | Nigeria |  | 130.2 | 111.4 | to | 149.0 |  | 104.6 | 76.0 | to | 133.2 |  | 710.1 | 595.7 | to | 824.5 |  | 303.0 | 139.0 | to | 466.9 |  |
|  | Oman |  | 15.3 | 11.1 | to | 19.5 |  | 11.4 | 6.4 | to | 16.4 |  | 60.8 | 50.2 | to | 71.4 |  | 12.5 | 7.3 | to | 17.7 |  |
|  | Pakistan |  | 69.1 | 55.9 | to | 82.3 |  | 56.1 | 40.9 | to | 71.3 |  | 300.8 | 241.7 | to | 359.9 |  | 109.4 | 62.5 | to | 156.4 |  |
|  | Palau |  | 19.0 | 14.8 | to | 23.2 |  | 18.1 | 10.9 | to | 25.3 |  | - | - |  | - |  | - | - |  | - |  |
|  | Panama |  | 18.2 | 13.8 | to | 22.6 |  | 16.2 | 9.0 | to | 23.4 |  | 46.4 | 37.9 | to | 54.9 |  | 49.5 | 36.4 | to | 62.6 |  |
|  | Papua New Guinea |  | 53.2 | 42.2 | to | 64.2 |  | 45.8 | 32.0 | to | 59.6 |  | 243.8 | 202.2 | to | 285.3 |  | 175.3 | 84.0 | to | 266.5 |  |
|  | Paraguay |  | 26.3 | 21.9 | to | 30.7 |  | 19.0 | 11.8 | to | 26.2 |  | 90.3 | 76.4 | to | 104.3 |  | 40.3 | 17.8 | to | 62.9 |  |
|  | Peru |  | 26.8 | 21.0 | to | 32.6 |  | 21.0 | 12.4 | to | 29.6 |  | 104.8 | 84.9 | to | 124.7 |  | 64.8 | 35.4 | to | 94.2 |  |
|  | Philippines |  | 28.6 | 22.8 | to | 34.4 |  | 20.4 | 13.0 | to | 27.8 |  | 113.3 | 95.2 | to | 131.4 |  | 87.0 | 56.2 | to | 117.8 |  |
|  | Poland |  | 6.5 | 4.9 | to | 8.1 |  | 6.1 | 3.7 | to | 8.5 |  | - | - |  | - |  | - | - |  | - |  |
|  | Romania |  | 18.4 | 14.2 | to | 22.6 |  | 15.2 | 9.0 | to | 21.4 |  | - | - |  | - |  | - | - |  | - |  |
|  | Russian Federation |  | 14.9 | 11.7 | to | 18.1 |  | 12.2 | 7.4 | to | 17.0 |  | - | - |  | - |  | - | - |  | - |  |
|  | Rwanda |  | 104.4 | 89.4 | to | 119.4 |  | 86.9 | 67.5 | to | 106.3 |  | 600.2 | 489.2 | to | 711.1 |  | 199.9 | 113.0 | to | 286.8 |  |
|  | Samoa |  | 16.4 | 12.8 | to | 20.0 |  | 15.2 | 9.8 | to | 20.6 |  | 161.6 | 134.7 | to | 188.4 |  | 111.8 | 80.9 | to | 142.6 |  |
|  | Senegal |  | 84.4 | 69.4 | to | 99.4 |  | 71.1 | 49.1 | to | 93.1 |  | 484.7 | 392.0 | to | 577.3 |  | 145.3 | 71.4 | to | 219.3 |  |
|  | Serbia |  | 10.3 | 8.3 | to | 12.3 |  | 8.6 | 5.2 | to | 12.0 |  | - | - |  | - |  | - | - |  | - |  |
|  | Seychelles |  | 10.0 | 7.4 | to | 12.6 |  | 8.1 | 4.5 | to | 11.7 |  | - | - |  | - |  | - | - |  | - |  |
|  | Sierra Leone |  | 180.5 | 158.1 | to | 202.9 |  | 141.0 | 103.0 | to | 179.0 |  | 1190.4 | 987.3 | to | 1393.5 |  | 435.7 | 207.2 | to | 664.2 |  |
|  | Slovak Republic |  | 8.5 | 6.3 | to | 10.7 |  | 8.1 | 4.5 | to | 11.7 |  | - | - |  | - |  | - | - |  | - |  |
|  | Solomon Islands |  | 23.0 | 18.6 | to | 27.4 |  | 19.7 | 13.3 | to | 26.1 |  | 114.3 | 98.6 | to | 130.0 |  | 50.3 | 30.9 | to | 69.7 |  |
|  | South Africa |  | 50.8 | 39.6 | to | 62.0 |  | 45.2 | 27.6 | to | 62.8 |  | 190.0 | 156.4 | to | 223.6 |  | 116.6 | 87.4 | to | 145.8 |  |
|  | Sri Lanka |  | 13.1 | 10.5 | to | 15.7 |  | 11.4 | 7.8 | to | 15.0 |  | 36.8 | 25.9 | to | 47.7 |  | 20.2 | 14.2 | to | 26.3 |  |
|  | St. Lucia |  | 13.3 | 10.5 | to | 16.1 |  | 10.9 | 6.3 | to | 15.5 |  | 37.2 | 30.8 | to | 43.7 |  | 17.8 | 12.8 | to | 22.9 |  |
|  | St. Vincent and the Grenadines |  | 16.2 | 12.2 | to | 20.2 |  | 14.3 | 8.1 | to | 20.5 |  | 30.5 | 25.3 | to | 35.6 |  | 19.8 | 13.2 | to | 26.3 |  |
|  | Sudan |  | 73.5 | 61.5 | to | 85.5 |  | 60.9 | 40.7 | to | 81.1 |  | 496.9 | 404.4 | to | 589.3 |  | 597.4 | 412.9 | to | 781.9 |  |
|  | Suriname |  | 29.7 | 23.7 | to | 35.7 |  | 22.6 | 13.2 | to | 32.0 |  | 61.0 | 50.1 | to | 71.8 |  | 35.9 | 22.2 | to | 49.7 |  |
|  | Swaziland |  | 78.7 | 60.9 | to | 96.5 |  | 68.2 | 46.4 | to | 90.0 |  | 180.5 | 145.8 | to | 215.2 |  | 108.2 | 77.2 | to | 139.3 |  |
|  | Syrian Arab Republic |  | 16.5 | 13.1 | to | 19.9 |  | 12.9 | 7.5 | to | 18.3 |  | 94.5 | 77.6 | to | 111.5 |  | 39.8 | 22.1 | to | 57.4 |  |
|  | Tajikistan |  | 75.3 | 65.9 | to | 84.7 |  | 63.2 | 43.4 | to | 83.0 |  | 116.6 | 97.2 | to | 135.9 |  | 63.0 | 38.8 | to | 87.2 |  |
|  | Tanzania |  | 86.5 | 73.7 | to | 99.3 |  | 72.1 | 54.9 | to | 89.3 |  | 577.6 | 477.8 | to | 677.5 |  | 223.4 | 120.8 | to | 326.0 |  |
|  | Thailand |  | 13.2 | 10.2 | to | 16.2 |  | 9.7 | 5.7 | to | 13.7 |  | 25.2 | 20.3 | to | 30.2 |  | 17.7 | 12.4 | to | 22.9 |  |
|  | Timor-Leste |  | 63.7 | 54.3 | to | 73.1 |  | 46.5 | 31.3 | to | 61.7 |  | 457.8 | 359.2 | to | 556.3 |  | 231.1 | 129.6 | to | 332.6 |  |
|  | Togo |  | 95.7 | 80.5 | to | 110.9 |  | 75.2 | 54.2 | to | 96.2 |  | 497.9 | 426.5 | to | 569.3 |  | 146.1 | 80.1 | to | 212.0 |  |
|  | Tonga |  | 14.4 | 11.6 | to | 17.2 |  | 13.1 | 8.7 | to | 17.5 |  | 38.7 | 31.7 | to | 45.7 |  | 48.1 | 37.4 | to | 58.8 |  |
|  | Trinidad and Tobago |  | 23.1 | 17.5 | to | 28.7 |  | 18.6 | 11.4 | to | 25.8 |  | 44.1 | 35.8 | to | 52.4 |  | 35.1 | 20.5 | to | 49.7 |  |
|  | Tunisia |  | 20.8 | 16.2 | to | 25.4 |  | 18.0 | 9.4 | to | 26.6 |  | 65.7 | 54.1 | to | 77.2 |  | 39.5 | 30.0 | to | 48.9 |  |
|  | Turkey |  | 24.1 | 18.7 | to | 29.5 |  | 20.5 | 11.3 | to | 29.7 |  | 37.2 | 29.5 | to | 45.0 |  | 27.1 | 17.7 | to | 36.5 |  |
|  | Turkmenistan |  | 52.5 | 43.7 | to | 61.3 |  | 43.7 | 26.5 | to | 60.9 |  | 55.8 | 44.4 | to | 67.3 |  | 49.3 | 36.1 | to | 62.5 |  |
|  | Tuvalu |  | 30.5 | 23.9 | to | 37.1 |  | 28.7 | 16.9 | to | 40.5 |  | - | - |  | - |  | - | - |  | - |  |
|  | Uganda |  | 95.6 | 80.2 | to | 111.0 |  | 75.3 | 58.7 | to | 91.9 |  | 283.3 | 229.9 | to | 336.7 |  | 130.3 | 64.1 | to | 196.5 |  |
|  | Ukraine |  | 13.4 | 11.2 | to | 15.6 |  | 11.2 | 7.0 | to | 15.4 |  | - | - |  | - |  | - | - |  | - |  |
|  | Uruguay |  | 11.9 | 9.1 | to | 14.7 |  | 10.0 | 5.2 | to | 14.8 |  | 20.5 | 16.5 | to | 24.5 |  | 13.3 | 9.6 | to | 17.1 |  |
|  | Uzbekistan |  | 46.7 | 37.7 | to | 55.7 |  | 38.3 | 24.7 | to | 51.9 |  | 24.8 | 19.9 | to | 29.7 |  | 17.6 | 13.1 | to | 22.1 |  |
|  | Vanuatu |  | 16.4 | 13.4 | to | 19.4 |  | 15.4 | 10.8 | to | 20.0 |  | 147.8 | 124.8 | to | 170.8 |  | 92.1 | 61.4 | to | 122.8 |  |
|  | Venezuela, RB |  | 16.3 | 12.5 | to | 20.1 |  | 12.7 | 6.7 | to | 18.7 |  | 67.0 | 56.2 | to | 77.8 |  | 41.4 | 25.6 | to | 57.2 |  |
|  | Vietnam |  | 22.8 | 19.0 | to | 26.6 |  | 16.0 | 10.0 | to | 22.0 |  | 85.8 | 52.6 | to | 118.9 |  | 52.7 | 36.5 | to | 68.9 |  |
|  | West Bank and Gaza |  | 22.4 | 17.8 | to | 27.0 |  | 17.2 | 10.6 | to | 23.8 |  | - | - |  | - |  | - | - |  | - |  |
|  | Yemen, Rep. |  | 73.1 | 61.9 | to | 84.3 |  | 51.3 | 34.5 | to | 68.1 |  | 398.6 | 337.0 | to | 460.2 |  | 58.7 | 15.2 | to | 102.2 |  |
|  | Zambia |  | 110.0 | 92.0 | to | 128.0 |  | 86.7 | 56.7 | to | 116.7 |  | 409.1 | 349.6 | to | 468.6 |  | 153.1 | 75.8 | to | 230.3 |  |
|  | Zimbabwe |  | 67.9 | 57.1 | to | 78.7 |  | 50.7 | 33.9 | to | 67.5 |  | 388.1 | 328.3 | to | 448.0 |  | 148.8 | 83.5 | to | 214.1 |  |
|  |  |  |  |  |  |  |  |  |  |  |  |  |  |  |  |  |  |  |  |  |  |  |

# BIBLIOGRAPHY

1. **Global Health Observatory Data Repository** [<http://apps.who.int/gho/data/node.main>]

2. **Global Health Expenditure Database: National Health Accounts (NHA)** [<http://apps.who.int/nha/database/DataExplorerRegime.aspx>]

3. **UN Data: A World of Information** [<http://data.un.org/Default.aspx>]

4. **International Human Development Indicators** [<http://hdrstats.undp.org/en/indicators/default.html>]

5. **Monitoring the Situation of Children and Women** [<http://www.childinfo.org/contact.html>]

6. **World Bank Data: World Development Indicators** [<http://databank.worldbank.org/data/home.aspx>]

7. **STAT Compiler Building Tables with DHS Data** [<http://statcompiler.com/>]

8. **The MacroData Guide - An International Social Science Resource: Fractionalization Data** [<http://www.nsd.uib.no/macrodataguide/set.html?id=16&sub=1>]

9. The World Bank: *World Development Report 2002: Building Institutions for Markets.* USA World Bank; Oxford University Press 2001.

10. **Composition of Macro Geographical (Continental) Regions, Geographical Sub-Regions, and Selected Economic and Other Groupings** [<http://unstats.un.org/unsd/methods/m49/m49regin.htm>]

11. **Every Women Every Child** [<http://www.everywomaneverychild.org/>]

12. **We Can End Poverty 2015: Millennium Development Goals: UN Summit 20-22 September 2010 New York: High-Level Planning Meeting of the General Assembly** [<http://www.un.org/en/mdg/summit2010/>]

13. Maternal Newborn & Child Health World Bank, Alliance for Health Policy and Systems Research University of St Gallen, Johns Hopkins University, Global Health Insights, London School of Hygiene and Tropical Medicine, United States Agency for International, World Health Organization: **Hope for 2015 and Beyond: Success Factors to Reduce Preventable Maternal and Child Deaths.** In *Book Hope for 2015 and Beyond: Success Factors to Reduce Preventable Maternal and Child Deaths* (Editor ed.^eds.). City: World Health Organizaton; 2013.

14. Sachs J: *Investing in Development: A Practical Plan to Achieve the Millennium Development Goals.* New York: United Nations Development Programme; 2005.

15. World Health Organization (WHO): *Everybody Business: Strengthening Health Systems to Improve Health Outcomes: Who’s Framework for Action.* Geneva, Switzerland; 2007.

16. Günther I, Fink G: **Water and Sanitation to Reduce Child Mortality: Te Impact and Cost of Water and Sanitation Infrastructure.** In *Book Water and Sanitation to Reduce Child Mortality: Te Impact and Cost of Water and Sanitation Infrastructure* (Editor ed.^eds.). City: Te World Bank Development Economics Prospects Group; 2011.

17. Stuckler D, Basu S, McKee M: **Drivers of Inequality in Millennium Development Goal Progress: A Statistical Analysis.** *PLoS Med* 2010, **7**.

18. Anyanwu JC, Erhijakpor AEO: **Health Expenditures and Health Outcomes in Africa.** *African Development Bank* 2009.

19. Issa H, Ouattara B: **The Effect of Private and Public Health Expenditure on Infant Mortality Rates: Does the Level of Development Matters?** In *Book The Effect of Private and Public Health Expenditure on Infant Mortality Rates: Does the Level of Development Matters?* (Editor ed.^eds.). City; 2005.

20. Farag M, Nandakumar AK, Wallack S, Hodgkin D, Gaumer G, Erbil C: **Health Expenditures, Health Outcomes and the Role of Good Governance.** *Int J Health Care Finance Econ* 2013, **13:**33–52.

21. Gottret P, Schieber G: **Health Financing Revisited: A Practioner's Guide.** In *Book Health Financing Revisited: A Practioner's Guide* (Editor ed.^eds.). City; 2006.

22. Bailey P, Paxton A, Lobis S, Fry D: **Measuring Progress Towards the MDG for Maternal Health: Including A Measure of the Health System’s Capacity to Treat Obstetric Complications.** *International Journal of Gynecology and Obstetrics* 2006, **93:**292–299.

23. Muldoon KA, Galway LP, Nakajima M, Kanters S, Hogg RS, Bendavid E, Mills EJ: **Health System Determinants of Infant, Child and Maternal Mortality: A Cross-Sectional Study of UN Member Countries.** *Globalization and Health* 2011, **7**.

24. Shandra CL, Shandra JM, London B: **World Bank Structural Adjustment, Water, and Sanitation: A Cross-National Analysis of Child Mortality in Sub-Saharan Africa.** *Organization & Environment* 2011, **24**.

25. Gakidou E, Oza S, Fuertes CV, Li AY, Lee DK, Sousa A, Hogan MC, Hoorn SV, Ezzati M: **Improving Child Survival Through Environmental and Nutritional Interventions: The Importance of Targeting Interventions Toward the Poor.** *JAMA* 2007, **298**.

26. Mcguire JW: **Basic Health Care Provision and Under-5 Mortality: A Cross-National Study of Developing Countries.** *World Development* 2006, **34:**405–425.

27. Soto M: **Seeds of Light Inside the Gloom: Assessing the Effect of Foreign Aid on the Reduction of Child Mortality** In *Book Seeds of Light Inside the Gloom: Assessing the Effect of Foreign Aid on the Reduction of Child Mortality* (Editor ed.^eds.). City; 2011.

28. Goldhaber-Fiebert JD, Lipsitch M, Mahal AJ, Zaslavsky AM, Salomon JA: **Quantifying Child Mortality Reductions Related to Measles Vaccination.** *PLoS ONE* 2010, **5**.

29. Rajkumar AS, Swaroop V: **Public spending and outcomes: Does governance matter?** *Journal of Development Economics* 2004, **86:**96–111.

30. Nishiyama A: **Economic Growth and Infant Mortality in Developing Countries.** *European Journal of Development Research* 2011, **23:**630–647.

31. El-Jardali F, Jamal D, Abdallah A, Kassak K: **Human Resources for Health Planning and Management in the Eastern Mediterranean Region: Facts, Gaps and Forward Thinking for Research and Policy.** *Human Resources for Health* 2007, **5**.

32. Anand S, Bärnighausen T: **Human Resources and Health Outcomes: Cross-Country Econometric Study.** *Lancet* 2004, **364:**1603–1609.

33. Sattler C, Shandra JM: **Do Health Nongovernmental Organizations Improve Child Mortality?** *International Journal of Sociology* 2012, **42:**28–46.

34. Mukherjee N: **Party Systems and Human Well-Being.** *Party Politics* 2011, **1**.

35. Chauvet L, Gubert F, Mesplé-Somps S: **Aid, Remittances, Medical Brain Drain and Child Mortality: Evidence Using Inter and Intra-Country Data.** *The Journal of Development Studies* 2012**:**1–18.

36. Farahani M, Subramanian SV, Canning D: **The Effect of Changes in Health Sector Resources on Infant Mortality in the Short-Run and the Long-Run: A Longitudinal Econometric Analysis.** *Social Science & Medicine* 2009, **68:**1918–1925.

37. Castillo-Laborde C: **Human Resources for Health and Burden of Disease: An Econometric Approach.** *Human Resources for Health* 2011, **9**.

38. Powell-Jackson T, Basu S, Balabanova D, McKee M, Stuckler D: **Democracy and growth in divided societies: A health-inequality trap?** *Social Science & Medicine* 2011, **73:**33–41.

39. Rabe-Hesketh S, Skrondal A: *Multilevel and Longitudinal Modeling Using Stata.* College Station: Stata Press; 2012.

40. UNFPA U, WHO, World Bank: **"Trends in Maternal Mortality: 1990-2010".** In *Book "Trends in Maternal Mortality: 1990-2010"* (Editor ed.^eds.). City: WHO; 2012.

41. Griliches Z, Hausman JA: **Errors in variables in panel data.** *Journal of Economietrics* 1986, **31:**93-118.

42. StataCorp: **Stata Statistical Software: Release 12.** In *Book Stata Statistical Software: Release 12* (Editor ed.^eds.). City: StataCorp LP; 2011.

43. Duan N: **Smearing Estimate: A Nonparametric Retransformation method.** *J Am Stat Ass* 1983, **78:**605–610.
